# Associations of genetic risk, BMI trajectories, and the risk of non-small cell lung cancer: a population-based cohort study

**DOI:** 10.1186/s12916-022-02400-6

**Published:** 2022-06-06

**Authors:** Dongfang You, Danhua Wang, Yaqian Wu, Xin Chen, Fang Shao, Yongyue Wei, Ruyang Zhang, Theis Lange, Hongxia Ma, Hongyang Xu, Zhibin Hu, David C. Christiani, Hongbing Shen, Feng Chen, Yang Zhao

**Affiliations:** 1 Department of Biostatistics, School of Public Health, Nanjing Medical University, 101 Longmian Avenue, Nanjing, 211166 Jiangsu China; 2 Department of Environmental Health, Harvard T.H. Chan School of Public Health, Boston, MA 02115 USA; 3 Department of Public Health and Preventive Medicine, Kangda College of Nanjing Medical University, Lianyungang, 222000 China; 4 China International Cooperation Center for Environment and Human Health, Center for Global Health, Nanjing Medical University, Nanjing, 211166 Jiangsu China; 5 The Center of Biomedical Big Data and the Laboratory of Biomedical Big Data, Nanjing Medical University, Nanjing, 211166 Jiangsu China; 6 Section of Biostatistics, Department of Public Health, Faculty of Health and Medical Sciences, University of Copenhagen, ØsterFarimagsgade 5, 1353 Copenhagen, Denmark; 7 Department of Epidemiology, School of Public Health, Nanjing Medical University, Nanjing, 211166 Jiangsu China; 8 Jiangsu Key Lab of Cancer Biomarkers, Prevention and Treatment, Collaborative Innovation Center for Cancer Personalized Medicine, Nanjing Medical University, Nanjing, 211166 Jiangsu China; 9 Department of Critical Care Medicine, Wuxi People’s Hospital Affiliated to Nanjing Medical University, Wuxi, 214023 Jiangsu China; 10 Department of Medicine, Massachusetts General Hospital/Harvard Medical School, Boston, MA 02115 USA

**Keywords:** Body mass index, Trajectory, Genome-wide interaction study, Non-small cell lung cancer

## Abstract

**Background:**

Body mass index (BMI) has been found to be associated with a decreased risk of non-small cell lung cancer (NSCLC); however, the effect of BMI trajectories and potential interactions with genetic variants on NSCLC risk remain unknown.

**Methods:**

Cox proportional hazards regression model was applied to assess the association between BMI trajectory and NSCLC risk in a cohort of 138,110 participants from the Prostate, Lung, Colorectal, and Ovarian (PLCO) Cancer Screening Trial. One-sample Mendelian randomization (MR) analysis was further used to access the causality between BMI trajectories and NSCLC risk. Additionally, polygenic risk score (PRS) and genome-wide interaction analysis (GWIA) were used to evaluate the multiplicative interaction between BMI trajectories and genetic variants in NSCLC risk.

**Results:**

Compared with individuals maintaining a stable normal BMI (*n* = 47,982, 34.74%), BMI trajectories from normal to overweight (*n* = 64,498, 46.70%), from normal to obese (*n* = 21,259, 15.39%), and from overweight to obese (*n* = 4,371, 3.16%) were associated with a decreased risk of NSCLC (hazard ratio [HR] for trend = 0.78, *P* < 2×10^−16^). An MR study using BMI trajectory associated with genetic variants revealed no significant association between BMI trajectories and NSCLC risk. Further analysis of PRS showed that a higher GWAS-identified PRS (PRS_GWAS_) was associated with an increased risk of NSCLC, while the interaction between BMI trajectories and PRS_GWAS_ with the NSCLC risk was not significant (*P*_*s*PRS_= 0.863 and *P*_*w*PRS_= 0.704). In GWIA analysis, four independent susceptibility loci (*P* < 1×10^−6^) were found to be associated with BMI trajectories on NSCLC risk, including rs79297227 (12q14.1, located in SLC16A7, *P*_interaction_ = 1.01×10^−7^), rs2336652 (3p22.3, near CLASP2, *P*_interaction_ = 3.92×10^−7^), rs16018 (19p13.2, in CACNA1A, *P*_interaction_ = 3.92×10^−7^), and rs4726760 (7q34, near BRAF, *P*_interaction_ = 9.19×10^−7^). Functional annotation demonstrated that these loci may be involved in the development of NSCLC by regulating cell growth, differentiation, and inflammation.

**Conclusions:**

Our study has shown an association between BMI trajectories, genetic factors, and NSCLC risk. Interestingly, four novel genetic loci were identified to interact with BMI trajectories on NSCLC risk, providing more support for the aetiology research of NSCLC.

**Trial registration:**

http://www.clinicaltrials.gov, NCT01696968.

**Supplementary Information:**

The online version contains supplementary material available at 10.1186/s12916-022-02400-6.

## Background

Lung cancer is one of the most common cancers and a leading cause of cancer-related death worldwide [1, 2]. In 2018, there were 2.09 million new cases and 1.76 million deaths of lung cancer worldwide, accounting for 11.6% and 18.4% of all cancer cases and deaths, respectively [3]. In particular, non-small cell lung cancer (NSCLC), the most common type of lung cancer, accounts for approximately 85% of all lung cancer cases [4]. Due to the increasing burden of NSCLC, it is necessary to identify more potential risk factors associated with NSCLC so as to develop individualized prevention strategies.

Obesity, usually defined as body mass index (BMI) ≥ 30 kg/m^2^, is becoming an increasingly common global health problem [5]. The global prevalence of obesity in adults increased steadily between 1975 and 2016, from 3 to 11% in men and 6 to 15% in women [6]. Several epidemiological studies have demonstrated that a higher BMI is associated with a lower risk of NSCLC in European and Asian populations [2, 7], which was also confirmed by a recent meta-analyses with a sample size of 7,310,130 participants [8]. However, most of these studies only used BMI at a single time point instead of considering the role of longitudinal BMI trajectories across the life course. In addition, a number of studies have shown that BMI trajectory from normal weight to obesity was associated with the risk of multiple cancers, including prostate, colorectal, oesophageal, gastric cardia adenocarcinoma, and even lung cancer [9–11].

Although environmental risk factors (e.g. BMI) are the main risk factors for NSCLC [12], genetic susceptibility is also an important contributor [13]. The heritability of lung cancer in European and Asian populations is estimated to be 12–21% [14, 15]. Previous genome-wide association studies (GWAS) identified more than 80 susceptibility variants associated with lung cancer in European and Asian populations, mainly NSCLC, as it is the main type of lung cancer; however, these variants could only explain a small proportion of the overall genetic variance [16, 17]. Interestingly, there is accumulating evidence that gene-environment interactions may be responsible for the missing heritability of cancer and act together with environmental risk factors in the pathogenesis of cancer [18, 19].

However, it remained unclear whether there was evidence to support the joint association between BMI trajectories and genetic variants on NSCLC incidence. In this study, we comprehensively investigated the relationship between BMI trajectories and NSCLC risk in the Prostate, Lung, Colorectal, and Ovarian (PLCO) Cancer Screening Trial. In addition, we applied a genome-wide interaction analysis to further assess the effect of different BMI trajectories in participants stratified according to genetic variants on NSCLC risk, which can provide novel insights into the pathophysiology of NSCLC.

## Methods

### Study population

The PLCO Cancer Screening Trial is a population-based cohort study aimed to evaluate the accuracy and reliability of screening methods for prostate, lung, colorectal, and ovarian cancer, which randomly recruited 154,897 individuals aged 49–78 years from 10 centres in the USA between 1993 and 2001 [20]. Exclusion criteria included (i) personal history of cancer prior to trial entry (*n* = 11,803); (ii) individuals with missing BMI at any age (*n* = 3,504) or BMI < 15 or > 50 kg/m^2^ (*n* = 361); (iii) individuals failing to return or complete the baseline questionnaire (*n* = 669); (iv) individuals at enrolment with age < 50 years (*n* = 2); and (v) individuals with small cell lung cancer (*n* = 448). Ultimately, a total of 138,110 participants were retained for analysis. No included individuals had been diagnosed with lung cancer at the time of voluntarily joining the study. The diagnosis of NSCLC was histologically confirmed via medical record reviews, the National Death Index (for completeness), and self-reported annual questionnaires during follow-up [21]. This study was approved by the ethics committees of the PLCO consortium providers (PLCO-424). Additional information for the study subjects is presented in the Additional file 1: Appendix S1 [22].

### BMI and BMI trajectories ascertainment

Height (m) and body weight (kg) at age 20, 50, and enrolment were collected from self-recorded questionnaires completed by the participants in the PLCO study (https://cdas.cancer.gov/datasets/plco/90/). BMI at each age period was calculated using the formula body weight (kg)/height (m^2^). Individuals were classified according to their BMI in each age period according to the World Health Organization 2000 criteria: underweight (<18.5 kg/m^2^), normal weight (18.5–24.9 kg/m^2^), overweight (25.0–29.9 kg/m^2^), and obesity (>30 kg/m^2^) [23]. To assess the relationship between pre-diagnostic BMI changes, defined as BMI from the age of 20 or 50 to the entry, and the risk of NSCLC at age 20, 50, and entry, latent class growth model (LCGM) was used to identify longitudinal patterns of BMI change at three-time points during adulthood [24]. Specifically, the LCGM here was fitted using linear and quadratic polynomials with three to five trajectory categories (individuals per trajectory ≥ 1%), and the model with the highest number of fitting categories was selected using the Bayesian Information Criterion (BIC) method and the average posterior probability (AvePP) of each trajectory [25]. Detailed information for the calculation of BMI trajectories is provided in the Additional file 1: Appendix S1 [26, 27].

### Genotyping

The PLCO GWAS data were deposited in the database of Genotypes and Phenotypes (dbGaP, phs001286.v1.p1 and phs000336.v1.p1), including a total of 14,497 participants genotyped using Illumina Hap240, Hap300, and Hap550 [28, 29]. The use of the PLCO genetic datasets was approved by both the Internal Review Board of Nanjing Medical University and the dbGaP database administration (#21708 and #21643). Basic information on genotyping and imputation for PLCO GWAS data is shown in the Additional file 1: Appendix S1 [30–32]. Additional quality control procedures for individuals and single nucleotide polymorphisms (SNPs) levels are presented in the Additional file 1: Appendix S1. Ultimately, 13,365 individuals remained in the genetic analysis (Additional file 1: Table S1).

### Analysis of the interaction between the GWAS-based polygenic risk score (PRS) and BMI trajectories

Based on 81 previously reported GWAS SNPs associated with lung cancer in European and Asian populations [16, 17], and a strict quality control process, including (i) SNPs located within autosomal chromosomes; (ii) minor allele frequency (MAF) ≥ 0.05; (iii) call rate ≥ 95%; (iv) *P*-value for Hardy-Weinberg Equilibrium (HWE) among non-NSCLC individuals ≥ 1.0×10^−6^; (v) imputation INFO > 0.3; and (vi) a risk effect consistent with previous results, we identified 19 independent [linkage disequilibrium (LD), *r*^*2*^ < 0.5] GWAS-identified SNPs (Additional file 1: Table S2) to construct the simple-count PRS (*s*PRS) and weighted PRS (*w*PRS) [16, 17, 33]. The *s*PRS is equal to the number of risk alleles, which can be estimated as $sPRS=\sum \limits_{i=1}^I{G}_i$, where G_*i*_ (i.e. 0, 1, or 2) denotes the number of risk alleles of *i*th SNP. The *w*PRS was calculated using the formula: $wPRS=\sum \limits_{i=1}^I{\beta}_i{G}_i$, where *β*_*i*_ is the per allele ORs derived from previous studies [16, 17, 33]. Additional information on the analysis of the interaction between the PRS_GWAS_ and BMI trajectory is presented in the Additional file 1: Appendix S1.

### Genome-wide interaction analysis (GWIA)

GWIA was performed to test for the gene-environment interactions between genome-wide SNPs and BMI trajectories. The interaction was modelled by determining the multiplicative product of SNP genotype and BMI trajectories in the Cox proportional hazard regression model, adjusting for age, sex, race, family history of lung cancer, education, smoking status, personal history of diabetes, current marital status, study centre, and the first 10 principal components. For GWIA, the *P*-value of the interaction term < 1.0×10^−6^ was considered statistically significant [34]. Similar to the construction of PRS_GWAS_, the GWIA-based *s*PRS (*s*PRS_GWIA_) or *w*PRS (*w*PRS_GWIA_) was also calculated to evaluate the cumulative interaction effects with BMI trajectories, separately.

### Functional annotation

Functional annotation was conducted to explore the potential molecular roles of the GWIA-identified loci by (i) pinpointing the most likely candidate genes at the identified loci by identifying *cis*-expression quantitative trait loci (*cis*-eQTL) within no more than 1 Mb of each investigated SNP in the Genotype-Tissue Expression project (version 7.0, http://www.gtexportal.org/home/) database from multiple relevant tissues [35, 36] and (ii) using the Encyclopedia of DNA Elements [37], HaploReg (version 4.1) [38], and RegulomeDB (http://www.regulomedb.org/) to further assess the regulatory potential for variants of interest.

### Statistical analysis

Cox proportional hazards regression model was used to estimate the hazard ratio (HR) and 95% confidence intervals (CIs) between BMI trajectories and NSCLC risk with adjustments for age, sex, race, family history of lung cancer, education, smoking status, personal history of diabetes, current marital status, and study centre. The proportional hazard assumption was assessed by Schoenfeld residuals [39]. Further, continuous variables were adapted to conduct tests of linear trends. Individual follow-up time was defined as a period from entry until the time of NSCLC occurrence (diagnosis) or censoring defined as the exit of the study due to other causes or death, loss to follow-up, or the end of the study.

Interaction effects of PRS_GWAS_, PRS_GWIA_, or each GWIA-identified SNP with BMI trajectories were further investigated by adding multiplicative interaction terms in the Cox models with adjustment for the first 10 principal components. A cumulative incidence function was estimated using Kaplan-Meier technique to quantify the risk of developing NSCLC over time, stratified by GWIA-identified SNPs, and differences in the full time-to-event distributions between different BMI trajectory groups were compared by a log-rank test [40].

Subgroup analysis was performed to evaluate the heterogeneity of the association between BMI trajectories and NSCLC risk stratified by sex, smoking status, or histological type. Further, several sensitivity analyses were performed to assess the reliability of the primary results. One-sample Mendelian randomization (MR) analysis was also performed to access the causality between BMI trajectories and NSCLC risk, including inverse-variance-weighted (IVW), Mendelian randomization Egger (MR-Egger), and simple median method. *P* values (two-sided) < 0.05 were deemed significant. All analyses were performed using R 3.5.3 and PLINK 1.90 software. Additional information is presented in the Additional file 1: Appendix S1.

## Results

There were 138,110 individuals in the prospective cohort study (Table 1). In total, 2641 NSCLC patients with a mean age of 64.34 years (SD = 5.20) were confirmed, including 2343 (88.72%) whites and 298 (11.28%) non-whites (184 blacks, 32 Hispanics, 63 Asians, and 19 others) populations. Compared with non-NSCLC individuals, NSCLC was more common among participants who were male (HR = 0.61, 95% CI: 0.56 to 0.66, *P* < 2×10^−16^), older (HR = 1.06, 95% CI: 1.05 to 1.07, *P* < 2×10^−16^), non-Hispanic Blacks (HR = 1.58, 95% CI: 1.36 to 1.84, *P* = 2.32×10^−9^), and current (HR = 24.22, 95% CI: 20.93 to 28.03, *P* < 2×10^−16^) or ever smoker (HR = 6.94, 95% CI: 6.01 to 8.01, *P* < 2×10^−16^); had a family history of lung cancer (HR = 1.83, 95% CI: 1.66 to 2.03, *P* < 2×10^−16^); had a low level of education (HR = 0.63, 95% CI: 0.58 to 0.69, *P* < 2×10^−16^); had a history of diabetes (HR = 1.25, 95% CI: 1.09 to 1.44, *P* = 0.001); and were divorced, separated, or widowed (HR = 1.41, 95% CI: 1.30 to 1.54, *P* = 1.08×10^−14^).

**Table 1:** Characteristics of the study subjects

Variables	Total (*N*=138,110)	NSCLC (*N*=2641)	Non-NSCLC (*N*=135,469)	HR (95% CI)	*P*-value*
Age (years)^a^, Mean ± SD	62.56 ± 5.34	64.34 ± 5.20	62.53 ± 5.34	1.06 (1.05, 1.07)	< 2×10^−16^
Sex, *N* (%)					
Male	69,713 (50.48)	1646 (62.32)	68,067 (50.25)	Reference	
Female	68,397 (49.52)	995 (37.68)	67,402 (49.75)	0.61 (0.56, 0.66)	< 2×10^−16^
Race, *N* (%)					
White, non-Hispanic	122,404 (88.63)	2343 (88.72)	120,061 (88.63)	Reference	
Black, non-Hispanic	6868 (4.97)	184 (6.97)	6684 (4.93)	1.58 (1.36, 1.84)	2.32×10^−9^
Hispanic	2552 (1.85)	32 (1.20)	2520 (1.86)	0.70 (0.50, 1.00)	0.049
Asian	5133 (3.72)	63 (2.39)	5070 (3.74)	0.62 (0.48, 0.80)	2.09×10^−4^
Other	1153 (0.83)	19 (0.72)	1134 (0.84)	0.91 (0.58, 1.43)	0.673
Family history of lung cancer, *N* (%)					
Absent	119,147 (86.88)	2050 (78.21)	117,097 (87.05)	Reference	
Present	14,376 (10.48)	447 (17.05)	13,929 (10.35)	1.83 (1.66, 2.03)	< 2×10^−16^
Missing	3616 (2.64)	124 (4.73)	4443 (2.60)		
Education, *N* (%)					
HS or less	41,506 (30.05)	1007 (38.13)	40,499 (29.90)	Reference	
Post HS or some college	47,376 (34.30)	986 (37.33)	46,390 (34.24)	0.90 (0.80, 1.01)	0.065
College graduate or degree	48,968 (35.46)	645 (24.42)	48,323 (35.67)	0.63 (0.58, 0.69)	< 2×10^−16^
Missing	260 (0.19)	3 (0.12)	257 (0.19)		
BMI at age 20 (kg/m^2^), Mean ± SD	22.10 ± 3.01	22.11 ± 2.99	22.10 ± 3.01	1.00 (0.99, 1.02)	0.520
BMI at age 50 (kg/m^2^), Mean ± SD	25.85 ± 4.17	25.16 ± 3.70	25.87 ± 4.18	0.96 (0.95, 0.97)	5.66×10^−15^
BMI at baseline (kg/m^2^), Mean ± SD	27.30 ± 4.75	26.49 ± 4.43	27.32 ± 4.75	0.96 (0.95, 0.97)	3.65×10^−16^
Smoking status, *N* (%)					
Never	63,945 (46.30)	217 (8.22)	63,728 (47.04)	Reference	
Former	59,685 (43.22)	1365 (51.68)	58,320 (43.06)	6.94 (6.01, 8.01)	< 2×10^−16^
Current	14,464 (10.47)	1059 (40.10)	13,405 (9.89)	24.22 (20.93, 28.03)	< 2×10^−16^
Missing	16 (0.01)	0	16 (0.01)		
Personal history of diabetes, *N* (%)					
Absent	127,024 (91.97)	2398 (90.80)	124,626 (92.00)	Reference	
Present	10,426 (7.55)	221 (8.37)	10,205 (7.53)	1.25 (1.09, 1.44)	0.001
Missing	660 (0.48)	22 (0.83)	638 (0.47)		
Current marital status, *N* (%)					
Married or living with someone	105,276 (76.23)	1890 (70.96)	103,386 (76.32)	Reference	
Divorced, separated, or widowed	28,030 (20.30)	677 (26.35)	27,353 (20.19)	1.41 (1.30, 1.54)	1.08×10^−14^
Single, never married	4580 (3.32)	71 (2.56)	4509 (3.33)	0.91 (0.71, 1.15)	0.409
Missing	224 (0.15)	3 (0.13)	221 (0.16)		
Hormone replacement therapy (in female), *N* (%)					
Never	22,083 (32.31)	389 (39.10)	21,694 (32.19)	Reference	
Current	34,779 (50.82)	411 (41.31)	34,368 (50.99)	0.67 (0.58, 0.77)	1.09×10^−8^
Former	11,120 (16.26)	187 (18.79)	10,933 (16.22)	0.95 (0.80, 1.14)	0.600
Missing	353 (0.61)	8 (0.80)	345 (0.60)		
Study centre, *N* (%)					
1 = University of Colorado	11,852 (8.58)	178 (6.74)	11,674 (8.62)	Reference	
2 = Georgetown University	6294 (4.56)	111 (4.20)	6183 (4.56)	1.13 (0.89, 1.43)	0.310
3 = Pacific Health Research and Education Institute (Honolulu)	9362 (6.78)	176 (6.66)	9186 (6.78)	1.24 (1.01, 1.53)	0.045
4 = Henry Ford Health System	21,887 (15.85)	442 (16.74)	21,445 (15.83)	1.47 (1.24, 1.75)	1.35×10^−5^
5 = University of Minnesota	24,613 (17.82)	510 (19.31)	24,103 (17.79)	1.36 (1.15, 1.62)	3.83×10^−4^
6 = Washington University in St Louis	13,763 (9.96)	310 (11.74)	13,453 (9.93)	1.54 (1.28, 1.85)	4.60×10^−6^
8 = University of Pittsburgh	16,021 (11.60)	350 (13.25)	15,671 (11.57)	1.48 (1.23, 1.77)	2.31×10^−5^
9 = University of Utah	13,449 (9.74)	175 (6.63)	13,274 (9.80)	0.87 (0.70, 1.07)	0.180
10 = Marshfield Clinic Research Foundation	15,153 (10.97)	289 (10.94)	14,864 (10.97)	1.25 (1.04, 1.51)	0.017
11 = University of Alabama at Birmingham	5716 (4.14)	100 (3.79)	5616 (4.15)	1.44 (1.13, 1.84)	0.004

*Univariate cox proportional hazard regression model

^a^Age at the time of study enrolment

*NSCLC* non-small cell lung cancer, *BMI* body mass index, *HR* hazard ratio, *CI* confidence interval

**Fig. 1:**
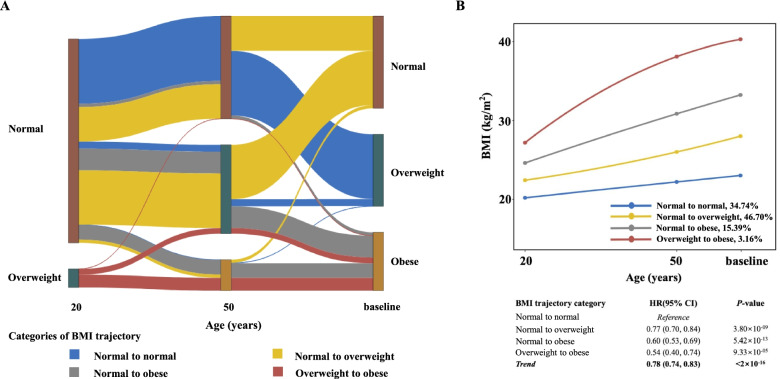
The latent class growth model of BMI trajectories in the PLCO study. **A** BMI changes for each participant in each trajectory group across three analysed age points (ages of 20 years, 50 years, and baseline). **B** Each trajectory was calculated at any of the three analysed age points (ages of 20 years, 50 years, and baseline). HR and 95% CI were estimated by Cox proportional hazards regression model with the adjustment for age, sex, race, family history of lung cancer, education, smoking, personal history of diabetes, current marital status, and study centre

No evidence of departure from the proportional hazard assumption in Cox models for NSCLC (*P* = 0.166) was found. Cox proportional hazards model showed that a higher BMI at 20 years, 50 years, and the time of enrolment (baseline) were associated with a decreased risk of NSCLC (HR = 0.88, *P* = 0.001; HR = 0.70, *P* < 2×10^−16^; HR = 0.75, *P* < 2×10^−16^, respectively), and similar findings were observed in categorical BMI (decreased risk in overweight and obesity, Additional file 1: Table S3). Further, we identified four distinct BMI trajectories by the latent class growth model (Fig. 1). Compared with participants with a stable normal BMI in their adulthood (*n* = 47,982, 34.74%), the risk of NSCLC decreased in participants who progressed from a normal BMI to an overweight BMI at baseline (*n* = 64,498, 46.70%, HR = 0.77, 95% CI: 0.70 to 0.84, *P* = 3.80×10^−9^), who progressed from a normal BMI to an obese BMI at baseline (*n* = 21,259, 15.39%, HR = 0.60, 95% CI: 0.53 to 0.69, *P* = 5.42×10^−13^), and who were overweight at the onset of adulthood and became obese at baseline (*n* = 4371, 3.16%, HR = 0.54, 95% CI: 0.40 to 0.74, *P* = 9.33×10^−5^). Interestingly, the NSCLC risk decreased gradually across all three BMI trajectories (HR for trend = 0.78, 95%CI: 0.74 to 0.83, *P* = 2×10^−16^) compared with subjects who maintained a normal BMI. Sensitivity analyses showed that the primary model retained a stable association between BMI trajectories and NSCLC risk (Additional file 1: Table S4). Furthermore, stratified analyses by sex, smoking status, and histological type showed almost no significant heterogeneity in the effect of age-specific BMI and BMI trajectories on NSCLC risk, although the *P* value for the heterogeneity test was less than 0.05 among those with BMI < 18.5 at baseline stratified by sex (Additional file 1: Figures S1-S3).

Nineteen GWAS-identified SNPs were used to construct the PRS and examine the potential effect of BMI trajectories on NSCLC risk according to the genetic variants. The characteristics of 13,365 individuals from the GWAS are shown in Additional file 1: Appendix S1. Nineteen GWAS-identified SNPs associated with lung cancer were used to construct the *s*PRS and *w*PRS (Additional file 1: Table S2). Furthermore, compared with the low tertiles of *s*PRS_GWAS_, the middle and high tertiles of *s*PRS_GWAS_ were associated with a higher probability of NSCLC (HR = 1.13, 95% CI: 1.12 to 1.59, *P* = 0.001; HR = 1.56, 95% CI: 1.34 to 1.82, *P* = 1.62×10^−8^, respectively) (Additional file 1: Table S5). Similar results were obtained for *w*PRS_GWAS_, indicating that a higher PRS_GWAS_ was associated with an increased risk of NSCLC. However, there was no significant interaction between BMI trajectories and PRS_GWAS_ with the NSCLC risk (*P*_*s*PRS_= 0.863 and *P*_*w*PRS_= 0.704; Additional file 1: Figure S4). Similar findings were observed for age-specific BMI (Additional file 1: Tables S6-S7).

**Fig. 2:**
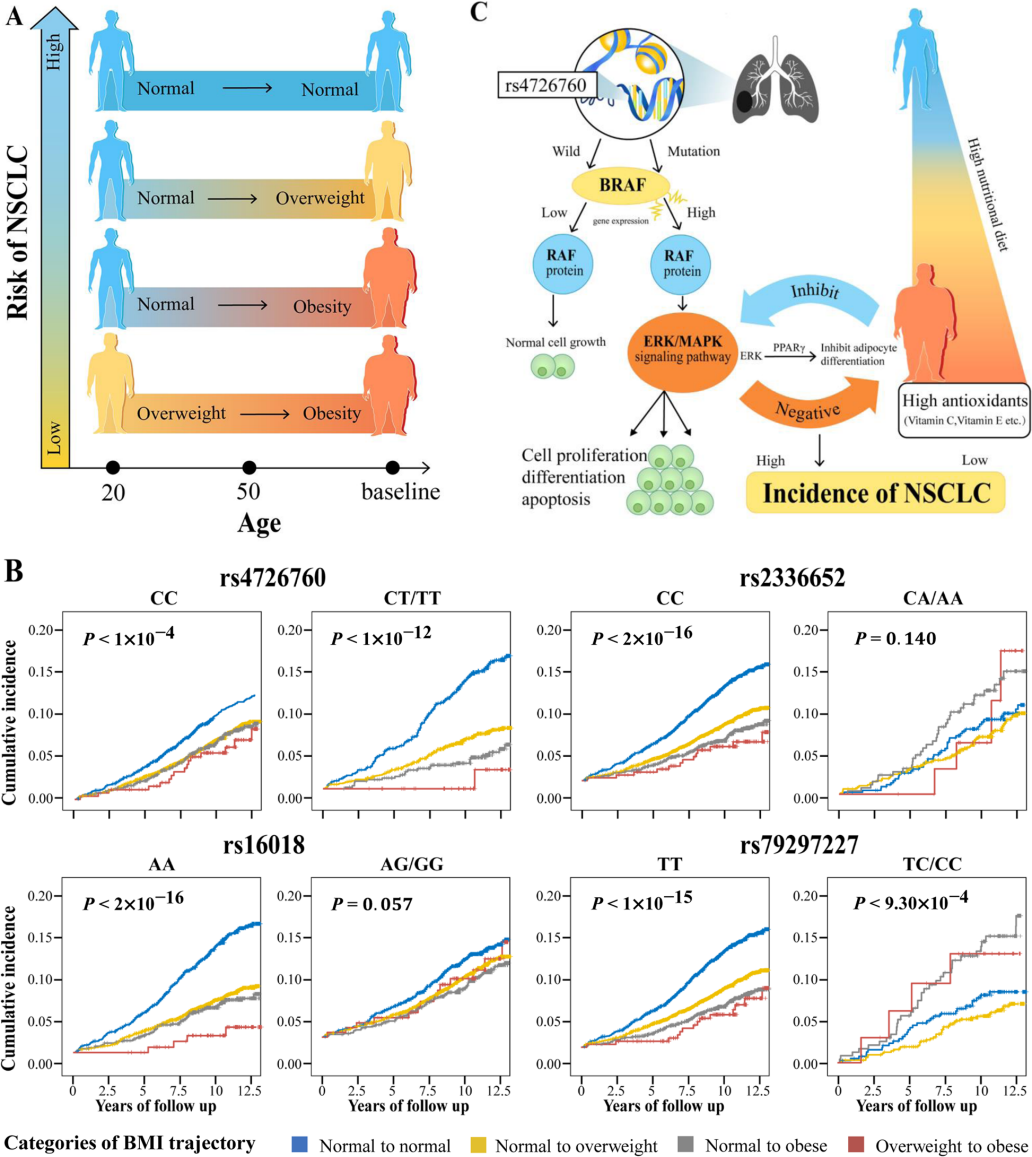
Stratifications analysis for the interaction effects between BMI trajectories and GWIA-identified SNPs on NSCLC risk. **A** The identified four BMI trajectories from the onset of adulthood to the baseline. **B** Cumulative incidence of NSCLC stratified by GWIA-identified SNPs. *P*-value was derived from the Log-rank test. **C** Pathway of the gene (BRAF)-BMI trajectories interaction effect on the risk of NSCLC

GWIA was subsequently performed to investigate the effect of the genome-wide interaction between each SNP and BMI trajectories on the NSCLC risk. A Manhattan plot was constructed to show the significant SNPs that interacted with BMI trajectories (Additional file 1: Figure S5). Four independent SNPs reached statistically suggestive significance [34] instead of genome-wide significance in GWIA, which were also confirmed in the bootstrap and permutation tests (Additional file 1: Table S8). Among the four SNPs, rs79297227 with the lowest *P* value (1.01×10^−7^) located in SLC16A7 (12q14.1) showed a statistically suggestively significant interaction with the BMI trajectories, and the remaining three SNPs, including rs2336652 near *CLASP2* (3p22.3, *P* = 3.92×10^−7^), rs16018 in *CACNA1A* (19p13.2, *P* = 3.92×10^−7^), and rs4726760 near *BRAF* (7q34, *P* = 9.19×10^−7^) interacted with the BMI trajectories in terms of the NSCLC risk. Similar results were obtained from the analysis stratified by genotype (Table 2). Figure 2B displays the cumulative incidence of NSCLC stratified by GWIA-identified SNPs by the log-rank test. In the sensitivity analysis, a significant interaction was observed between four SNPs and the BMI trajectories by additionally adjusting for occupation and family history of any cancer or performing other sensitivity analyses (almost *P* < 1.0×10^−4^, Additional file 1: Table S9). MR sensitivity analyses showed that the correlation direction between BMI trajectories and NSCLC risk was consistent with the above analysis, although no meaningful differences in these results were observed, with no evidence of directional pleiotropy (Additional file 1: Tables S10-S11). For the functional annotation, the search for cis-eQTLs at the four loci detected by GWIA showed that SNP rs4726760 at 7q34 was a strong cis-eQTL for *BRAF* (*P* = 0.011, *β* = 0.073) in the lung tissue. No cis-eQTL was found at the other three loci (rs16018, *P* = 0.070, *β* = 0.128; rs2336652, *P* = 0.854, *β* = −0.015; rs79297227, *P* = 0.376, *β* = −0.042) (Additional file 1: Figure S6A). SNP rs16018 is located on chromosome 19p13.2 in *calcium voltage-gated channel subunit alpha1 A* (*CACNA1A)*, which is a protein-coding gene involved in calcium channel regulation; SNP rs2336652 at 3p22.3 is located near *cytoplasmic linker-associated protein 2* (*CLASP2)*,which is significantly expressed in lung tissue and promotes the stability of microtubules; and SNP rs79297227 at 12q14.1 is located in the *solute carrier family 16 member 7* (*SLC16A7*), which is not only significantly expressed in lung tissues (Additional file 1: Figure S6B) but also expressed in various types of malignant tumours.

**Table 2:** Association between BMI trajectories and NSCLC risk stratified by the four susceptibility SNPs

SNP/Genotype	BMI trajectory				HR_*trend*_ (95% CI)	*P*_*trend*_	*P*_*interaction*_
	Normal BMI	Normal to overweight	Normal to obese	Overweight to obese			
rs79297227							1.01×10^−7^
TT							
NSCLC/Non-NSCLC	402/2947	481/5593	111/1772	14/268			
HR (95% CI)^a^	Reference	0.75 (0.65, 0.85)	0.53 (0.42, 0.67)	0.42 (0.24, 0.74)	0.74 (0.67, 0.81)	3.37×10^−10^	
TC/CC							
NSCLC/Non-NSCLC	30/353	39/583	34/199	4/29			
HR (95% CI)^a^	Reference	1.06 (0.62, 1.81)	2.54 (1.42, 4.53)	1.73 (0.49, 6.05)	1.49 (1.14, 1.94)	0.003	
rs2336652							3.92×10^−7^
CC							
NSCLC/Non-NSCLC	408/3031	465/5692	115/1810	13/282			
HR (95% CI)^a^	Reference	0.72 (0.63, 0.84)	0.54 (0.43, 0.68)	0.34 (0.18, 0.62)	0.73 (0.66, 0.80)	6.51×10^−11^	
CA/AA							
NSCLC/Non-NSCLC	40/398	64/734	32/229	4/31			
HR (95% CI)^a^	Reference	0.98 (0.64, 1.51)	1.85 (1.09, 3.12)	2.05 (0.69, 6.10)	1.33 (1.04, 1.70)	0.025	
rs16018							3.92×10^−7^
AA							
NSCLC/Non-NSCLC	252/1645	233/3103	63/983	4/153			
HR (95% CI)^a^	Reference	0.58 (0.48, 0.70)	0.49 (0.36, 0.66)	0.12 (0.03, 0.47)	0.64 (0.56, 0.73)	8.56×10^−11^	
AG/GG							
NSCLC/Non-NSCLC	197/1789	299/3328	84/1058	14/160			
HR (95% CI)^a^	Reference	0.94 (0.78, 1.14)	0.81 (0.62, 1.08)	0.87 (0.50, 1.52)	0.92 (0.82, 1.04)	0.187	
rs4726760							9.19×10^−7^
CC							
NSCLC/Non-NSCLC	308/2528	412/4656	121/1433	16/235			
HR (95% CI)^a^	Reference	0.88 (0.76, 1.04)	0.86 (0.69, 1.09)	0.57 (0.33, 0.98)	0.90 (0.81, 0.99)	0.030	
CT/TT							
NSCLC/Non-NSCLC	126/811	110/1589	23/522	1/68			
HR (95% CI)^a^	Reference	0.47 (0.35, 0.62)	0.24 (0.14, 0.40)	0.13 (0.02, 0.95)	0.48 (0.39, 0.59)	2.07×10^−11^	

^a^Cox proportional hazard regression model adjusted age, sex, race, family history of lung cancer, education, smoking, personal history of diabetes, current marital status, study centre, and first10 principal component

*BMI* body mass index, *NSCLC* non-small cell lung cancer, *SNPs* single nucleotide polymorphisms, *HR* hazard ratio, *CI* confidence interval

**Fig. 3:**
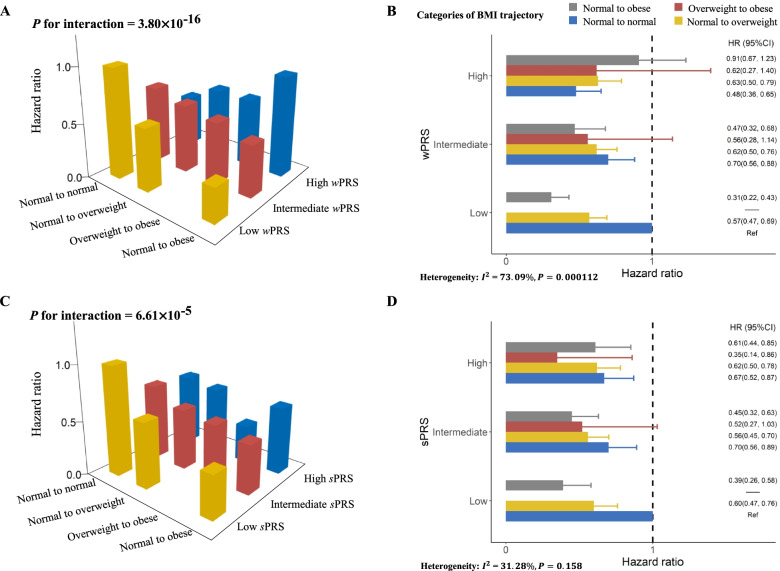
Interaction analysis and stratification analysis of BMI trajectories and the PRS constructed by four GWIA-identified SNPs on NSCLC risk. **A**, **B***w*PRS_GWIA_ were weighted according to the strength of their association with lung cancer. **C**, **D***s*PRS_GWIA_ were calculated by simple counting. *P* value for interaction was derived from multivariate-adjusted Cox proportional hazards regression model. PRS, polygenic risk score; GWIA, genome wide interaction analysis; SNP, single nucleotide polymorphism; HR, hazard ratio; CI, confidence interval

GWIA-based PRS of the four SNPs above was constructed to evaluate the cumulative interaction with BMI trajectories on NSCLC risk (Fig. 3). Although a significant association was identified between BMI trajectories and a higher NSCLC risk among the individuals with high tertiles of *w*PRS_GWIA_ (HR for trend =1.30, 95% CI = 1.10–1.54), interestingly, BMI trajectories were also associated with a decreased risk of NSCLC among individuals with a low (0.54, 0.47–0.62) or intermediate tertiles of *w*PRS_GWIA_ (0.85, 0.72–0.99), indicating an obvious interaction between the GWIA-based *w*PRS_GWIA_ and BMI trajectories. Similar findings were observed for age-specific BMI (Additional file 1: Table S12). The interaction between BMI trajectories and PRS_GWIA_ with the NSCLC risk was significant (*P*_*s*PRS_ = 6.61×10^−5^ and *P*_*w*PRS_ = 3.80×10^−16^; Additional file 1: Figure S4). In addition, individuals with low or intermediate tertiles of *w*PRS_GWIA_ experienced a gradually decreased cancer risk across the BMI trajectories from normal to normal, normal to overweight, overweight to obese, and normal to obese, while the high tertiles of *w*PRS_GWIA_ were just the opposite after adjustment for age, sex, race, family history of lung cancer, education, smoking, personal history of diabetes, current marital status, study centre, and first 10 principal components (Fig. 3A, B). Stratification analyses for *w*PRS_GWIA_ showed that associations between BMI trajectories and NSCLC risk were heterogeneous (*I*^*2*^ = 73.09%, *P* for heterogeneity < 0.001, Fig. 3B). Similar results were also observed in *s*PRS_GWIA_ (Additional file 1: Figure S4CD, Table S13).

## Discussion

In this multi-centre study, four distinct trajectories of BMI were identified during adulthood, finding that subjects who progressed from a normal BMI at the onset of adulthood to overweight or obesity at baseline (compared to maintaining a stable BMI) had a lower risk of developing NSCLC in this PLCO cohort (Fig. 2A). In addition, interaction analysis provided evidence that the association between BMI trajectories and NSCLC risk slightly differed according to genetic variation at SNPs rs4726760, rs16018, rs2336652, and rs79297227.

The results of this study suggested that the BMI trajectory from normal weight to overweight or obesity was associated with protective effects against NSCLC development, which was consistent with previous epidemiology studies [1, 2, 41–43]. Several hypotheses have been postulated to explain the relationship between leanness and a higher risk of lung cancer. For example, smoking, as a dominant risk factor for lung cancer, usually leads to lower body weight, which may explain the observed inverse BMI-lung cancer association. However, several large prospective studies show a negative association between BMI and lung cancer risk, and this association persists after excluding up to 10 years of follow-up, suggesting that it is not entirely due to smoking [44]. Moreover, never-smokers were more likely to have a stable normal BMI trajectory according to a stratified analysis of smoking status, although never-smokers in each BMI trajectory group accounted for about 50% of our analysis. Likewise, it has been suggested that weight loss represents a preclinical event prior to the clinical manifestation of lung cancer [45]. However, our sensitivity analysis suggested that BMI trajectories resulting in overweight or obesity were associated with a lower risk of lung cancer, even excluding patients who developed the disease during the first, second, or fourth year of follow-up. Interestingly, interaction analysis of PRS_GWIA_ with BMI trajectories on NSCLC risk indicated that BMI progressed from normal to overweight or obesity was associated with higher NSCLC risk among individuals with the high tertiles of *w*PRS_GWIA_ or *s*PRS_GWIA_. Specifically, they experienced a gradually increased NSCLC risk across the BMI trajectories from normal to normal, normal to overweight, overweight to obese, and normal to obese, although the low or intermediate tertiles of *w*PRS_GWIA_ or *s*PRS_GWIA_ were just the opposite (Fig. 3). In addition, those identified SNPs were located in or near genes that might be involved in biological pathways leading to lung cancer. The gene *BRAF* near rs4726760 provides instructions for making a protein that helps transmit chemical signals from outside the cell to the nucleus. This protein is a component of the extracellular signal-regulated kinase (ERK)/mitogen-activated protein kinase (MAPK) pathway, which regulates several important cell functions including cellular proliferation, differentiation, migration, and apoptosis. Chemical signalling through this pathway is essential for normal development before birth. *BRAF* also is an oncogene. When mutated, oncogenes have the potential to cause normal cells to become cancerous [46]. *BRAF* mutations are seen in 3–5% of NSCLC cases [47]. It is generally believed that obese people eat nutrient-rich foods, and studies have found that nutrients (antioxidants) can significantly inhibit the MAPK signalling pathway to reduce the inflammation response related to the risk of cancer [48]. The MAPK pathway plays an important role in the differentiation of adipocytes [49], and ERK is essential for the transcription of gene CCATT/enhancer binding protein α/β/δ and peroxisome proliferator-activated receptor gamma (PPARγ), key factors of adipocyte differentiation. When the ERK signalling pathway is activated, PPARγ is phosphorylated and transcriptional activity is reduced, which inhibits adipocyte differentiation [50]. Decreased adipocyte differentiation reduces the accumulation of adipocytes, thereby reducing the incidence of inflammation that may be related to pathological obesity (Fig. 2C).

The SNP rs16018, a member of the family of voltage-gated calcium channels, is located in the gene *CACNA1A* which is upregulated in numerous types of cancer including lung cancer [51]. The roles of calcium channels in various cell functions including mitogenesis, cell proliferation, differentiation, inflammation, and metastasis are well recognized [52]. Through calmodulin, intracellular calcium (Ca^2+^) levels regulate many different kinases, phosphatases, cyclases, esterases, and ion channels. Increased intracellular Ca^2+^ levels are correlated with cell proliferation, leading to inflammation and promoting carcinogenesis [51]. Subjects with a higher BMI may have sufficient nutritional status, and current studies have demonstrated that people with higher intake of nutrients (e.g. high dietary calcium) can modulate circulating calcitriol, thereby regulating intracellular Ca^2+^ levels [53], maintaining the balance of intracellular and extracellular Ca^2+^ concentrations and reducing the risk of lung cancer.

The SNP rs2336652, located near *CLASP2*, interacts with cytoplasmic linker protein, binds to microtubules, and has microtubule-stabilizing effects [54]. Increasing microtubule instability may cause genetic instability, and altered expression of *CLASP*2 may induce genetic instability and contribute to the development of lung cancer [55]. The variant rs79297227 is associated with the expression of *SLC16A7*. The *SLC16A* family of monocarboxylate transporters is a subfamily of solute carriers that transport monocarboxylate molecules, including L-lactate and pyruvate, across cell membranes [56]. Aberrant expression of *SLC16A* gene family members occurs in various types of malignant tumours and regulates cell migration, invasion, and proliferation [57–59].

MR analysis revealed non-significant associations between genetic polymorphisms affecting BMI and NSCLC. Although MR is considered a powerful tool to infer causality from nature’s randomization, it cannot completely avoid bias and confounders; thus, the results of MR studies warrant a cautious interpretation [60]. For example, BMI is strongly affected by smoking status, age, sex, and ethnicity [61]. However, confounding could not result in the genetic variant, and it is possible that attenuation of a protective effect against NSCLC has been caused by adjustment for mediators actually along the causal pathway or associated with collider bias [62]. In the end, the use of BMI variants in MR as proxies for BMI trajectories had inherent limitations due to the lack of previous GWAS studies on BMI trajectories, and insufficient PLCO genetic data despite the large sample in the PLCO cohort.

Our study had several strengths. First, this study was performed in a multi-centre, large sample size cohort. Second, we not only investigated the association between BMI trajectories and the NSCLC risk but also evaluated the interaction between BMI trajectories and genetic variants in the development of NSCLC. Third, we identified four novel and functionally plausible GWIA-based SNPs, which located near genes that paly critical roles in cell growth, differentiation, and inflammation and were mechanistically linked to BMI and NSCLC genesis. However, limitations of this study have also been identified. Similar to nearly all epidemiologic study on the subject, BMI at age 20 and 50 were obtained from individual’s self-report. However, that information was obtained before the subsequent development of the outcomes of interest, so recall bias could not have been operative. Second, a substantial number of exclusions could limit generalizability, while it constrained our study cohort to those with complete data available that should help mitigate against threats to internal validity. Third, residual for unmeasured confounding cannot be excluded even exhaustive adjustment was performed in the multivariable analyses. And conclusions from further Mendelian randomization, which purportedly provides a methodologic approach for causality inference, should also be treated with caution. Fourth, our findings have not been validated by other larger-sample epidemiological studies, especially the limited sample size of the PLCO GWAS data. Finally, additional functional studies are warranted to elucidate the mechanisms underlying the effects of these loci and BMI trajectories interactions on NSCLC risk.

## Conclusions

Our study found that genetic susceptibility may modify the effect of BMI trajectories on the development of NSCLC by regulating cell growth, differentiation and inflammation. Further larger or multi-ethnicity studies should be conducted to validate our findings.

## Supplementary Information

**Additional file 1: Appendix S1.** Supplementary methods. **Table S1.** Characteristics of the study subjects for GWAS. **Table S2.** Summary of the 19 GWAS-identified SNPs associated with lung cancer. **Table S3.** Association between age-specific BMI and NSCLC risk. **Table S4.** Sensitivity analysis for the association between BMI trajectory and NSCLC risk. **Table S5.** Association of *s*PRS_GWAS_ and *w*PRS_GWAS_ with NSCLC risk. **Table S6.** Interaction analysis between age-specific BMI and the *s*PRS_GWAS_. **Table S7.** Interaction analysis between age-specific BMI and the *w*PRS_GWAS_. **Table S8.** Summary of four independent SNPs identified by GWIA. **Table S9.** Sensitivity analyses for the interaction between BMI trajectory and rs79297227, rs2336652, rs16018 and rs79297227. **Table S10.** Single-nucleotide polymorphisms used as instrumental variables in the multivariable Mendelian randomization analyses of BMI trajectory. **Table S11.** Sensitivity analysis of the relationship between BMI trajectory and lung cancer using one-sample Mendelian randomization. **Table S12.** Interaction analysis between age-specific BMI/BMI trajectories and the *s*PRS_GWIA_. **Table S13.** Interaction analysis between age-specific BMI/BMI trajectories and the *w*PRS_GWIA_. **Figure S1.** Stratification analysis for age-specific BMI and BMI trajectory on NSCLC risk by sex. **Figure S2.** Stratification analysis for age-specific BMI and BMI trajectory on NSCLC risk by smoking status. **Figure S3.** Stratification analysis for age-specific BMI and BMI trajectory on NSCLC risk by histological type. **Figure S4.** Association of multivariate-adjusted NSCLC risk with BMI trajectories according to PRS_GWIA_ categories. **Figure S5.** Circle Manhattan Plot for interaction analysis between SNPs and BMI trajectory in regard to NSCLC risk. **Figure S6.** Analysis of the four loci and related gene expression in lung tissue.

## Data Availability

The PLCO phenotypic data and GWAS data analysed during the current study are available from the corresponding author on reasonable request.

## Abbreviations

BMI: Body mass index
CI: Confidence interval
DNA: Deoxyribonucleic acid
eQTL: Expression quantitative trait loci
GWAS: Genome-wide association study
GWIA: Genome-wide interaction analysis
HR: Hazard ratio
HWE: Hardy-Weinberg equilibrium
LD: Linkage disequilibrium
MAF: Minor allele frequency
MAPK: Mitogen-activated protein kinase
MR: Mendelian randomization
NSCLC: Non-small cell lung cancer
PLCO: Prostate, lung, colorectal, and ovarian
PRS: Polygenic risk score
PRS_GWAS_: Polygenic risk score constructed by 19 previous GWAS-identified SNPs
PRS_GWIA_: Polygenic risk score constructed by 4 GWIA-identified SNPs
SCLC: Small cell lung cancer
SD: Standard deviation
SNP: Single nucleotide polymorphism
*s*PRS: Simple-count polygenic risk score
*s*PRS_GWAS_: Simple-count polygenic risk score constructed by 19 previous GWAS-identified SNPs
*s*PRS_GWIA_: Simple-count polygenic risk score constructed by 4 GWIA-identified SNPs
*w*PRS: Weighted polygenic risk score
*w*PRS_GWAS_: Weighted polygenic risk score constructed by 19 previous GWAS-identified SNPs
*w*PRS_GWIA_: Weighted polygenic risk score constructed by 4 GWIA-identified SNPs
