# Supplementary material for: Associations of genetic risk, BMI trajectories, and the risk of non-small cell lung cancer: a population-based cohort study
Source: BMC Med. 2022 Jun 6;20:203. doi: 10.1186/s12916-022-02400-6 (PMC9169327; doi:10.1186/s12916-022-02400-6)
Supplement: Supplementary file 1 — Additional file 1: Appendix S1. Supplementary methods. Table S1. Characteristics of the study subjects for GWAS. Table S2. Summary of the 19 GWAS-identified SNPs associated with lung cancer. Table S3. Association between age-specific BMI and NSCLC risk. Table S4. Sensitivity analysis for the association between BMI trajectory and NSCLC risk. Table S5. Association of sPRSGWAS and wPRSGWAS with NSCLC risk. Table S6. Interaction analysis between age-specific BMI and the sPRSGWAS. Table S7. Interaction analysis between age-specific BMI and the wPRSGWAS. Table S8. Summary of four independent SNPs identified by GWIA. Table S9. Sensitivity analyses for the interaction between BMI trajectory and rs79297227, rs2336652, rs16018 and rs79297227. Table S10. Single-nucleotide polymorphisms used as instrumental variables in the multivariable Mendelian randomization analyses of BMI trajectory. Table S11. Sensitivity analysis of the relationship between BMI trajectory and lung cancer using one-sample Mendelian randomization. Table S12. Interaction analysis between age-specific BMI/BMI trajectories and the sPRSGWIA. Table S13. Interaction analysis between age-specific BMI/BMI trajectories and the wPRSGWIA. Figure S1. Stratification analysis for age-specific BMI and BMI trajectory on NSCLC risk by sex. Figure S2. Stratification analysis for age-specific BMI and BMI trajectory on NSCLC risk by smoking status. Figure S3. Stratification analysis for age-specific BMI and BMI trajectory on NSCLC risk by histological type. Figure S4. Association of multivariate-adjusted NSCLC risk with BMI trajectories according to PRSGWIA categories. Figure S5. Circle Manhattan Plot for interaction analysis between SNPs and BMI trajectory in regard to NSCLC risk. Figure S6. Analysis of the four loci and related gene expression in lung tissue. [file 12916_2022_2400_MOESM1_ESM.docx]

**Supplementary materials for:**

***“Associations of genetic risk, BMI trajectories and the risk of non-small cell lung cancer: a population-based cohort study”***

**Contents**

[**Appendix S1. Supplementary methods.** 3](#_Toc102986657)

[**Table S1. Characteristics of the study subjects for GWAS.** 8](#_Toc102986658)

[**Table S2. Summary of the 19 GWAS-identified SNPs associated with lung cancer.** 11](#_Toc102986659)

[**Table S3. Association between age-specific BMI and NSCLC risk.** 12](#_Toc102986660)

[**Table S4. Sensitivity analysis for the association between BMI trajectory and NSCLC risk.** 13](#_Toc102986661)

[**Table S5. Association of *s*PRS_GWAS_ and *w*PRS_GWAS_ with NSCLC risk.** 15](#_Toc102986662)

[**Table S6. Interaction analysis between age-specific BMI and the *s*PRS_GWAS_.** 16](#_Toc102986663)

[**Table S7. Interaction analysis between age-specific BMI and the *w*PRS_GWAS_.** 17](#_Toc102986664)

[**Table S8. Summary of four independent SNPs identified by GWIA.** 18](#_Toc102986665)

[**Table S9. Sensitivity analyses for the interaction between BMI trajectory and rs79297227, rs2336652, rs16018 and rs79297227.** 19](#_Toc102986666)

[**Table S10. Single-nucleotide polymorphisms were used as instrumental variables in the multivariable Mendelian randomization analyses of BMI trajectory.** 21](#_Toc102986667)

[**Table S11. Sensitivity analysis of the relationship between BMI trajectory and lung cancer using one-sample Mendelian randomization.** 22](#_Toc102986668)

[**Table S12. Interaction analysis between age-specific BMI/BMI trajectories and the *w*PRS_GWIA_.** 23](#_Toc102986669)

[**Table S13. Interaction analysis between age-specific BMI/BMI trajectories and the *s*PRS_GWIA_.** 24](#_Toc102986670)

[**Figure S1. Stratification analysis for age-specific BMI and BMI trajectory on NSCLC risk by sex.** 25](#_Toc102986671)

[**Figure S2. Stratification analysis for age-specific BMI and BMI trajectory on NSCLC risk by smoking status.** 26](#_Toc102986672)

[**Figure S3. Stratification analysis for age-specific BMI and BMI trajectory on NSCLC risk by histological type.** 27](#_Toc102986673)

[**Figure S4. Association of multivariate-adjusted NSCLC risk with BMI trajectories according to PRS_GWIA_ categories.** 28](#_Toc102986674)

[**Figure S5. Circle Manhattan Plot for interaction analysis between SNPs and BMI trajectory regarding NSCLC risk.** 29](#_Toc102986675)

[**Figure S6. Analysis of the four loci and related gene expression in lung tissue.** 30](#_Toc102986676)

# **Appendix S1. Supplementary methods.**

*Study participants*

The PLCO Cancer Screening Trial is a large, randomized, two-arm trial deigned to evaluate the efficacy of screening methods for prostate, lung, colorectal and ovarian cancer to reduce cancer mortality. Briefly, the participants aged 49-78 were enrolled from 1993 to 2001. At entry, participants were randomly assigned to the intervention arm (*n* = 77,444) and the control arm (*n* = 77,453). All participants completed a detailed questionnaire at enrollment including information on demographics, diet, cancer risk factors, family history and personal cancer history. The subjects were followed until 31 December 2009. The PLCO study was approved by the institutional review boards of the U.S. National Cancer Institute and the 10 PLCO screening. An informed consent agreement for participation in the research study was also obtained. Access to PLCO phenotypic data was granted under Project Number PLCO-424 and approved by the ethics committees of Nanjing Medical University (Jiangsu, China).

*BMI trajectory ascertainment*

We used the latent class growth model to identify BMI trajectories for all individuals using the BMI at age of 20, 50, and enrolment (baseline, over 50 years old). The optimal model was selected based on the following criteria: (i) for each trajectory group, obtaining a close correspondence between the estimated probability of group membership and the proportion assigned to that group based on the posterior probability of group membership; (ii) ensuring that the average of the posterior probabilities of group membership for individuals assigned to each group exceeded a minimum threshold of 0.7; (iii) establishing that the odds of correct classification based on the posterior probabilities of group membership exceeded a minimum threshold of 5; and (iv) observing reasonably tight confidence intervals around estimated group membership probabilities.

*Genotyping*

Sequential blood samples were collected from participants assigned to the screening arm. Ninety-three percent of participants assigned to the screening arm provided a baseline blood sample. In the observational (control) arm, buccal cells were collected via mail using the “swish-and-spit” protocol, and the participation rate was 65%. A detailed description of the PLCO study is available online (http://dcp.cancer.gov/plco).

*Imputation*

Detailed descriptions of the imputation procedures used in this study have been published in previous studies. Briefly, qualified genotypes for each chromosome were phased with SHAPEIT2. Non-genotyped SNPs were imputed based on haplotypes derived from the 1000 Genomes Project and the Haplotype Reference Consortium (HRC) reference panel. In this study, we imputed the genotyping data with IMPUTE2 software (<https://mathgen.stats.ox.ac.uk/impute/impute_v2.html>) according to the 1000 Genomes Project (phase 1 release v3).

*Quality control (QC)*

Criteria at the single nucleotide polymorphism (SNP) level were mapped to autosomal chromosomes, minor allele frequency (MAF) ≥ 0.05, *P* value for Hardy-Weinberg equilibrium (HWE) among non-NSCLC individuals ≥ 1.0×10^-6^, genotype missing rate ≤ 0.05, and SNPs with a high imputation quality score ≥ 0.3. After imputation and quality control analysis, a total of 13,365 unduplicated individuals (**Table S1**) with 4,250,895 SNPs were used for analysis.

*Analysis of the interaction between the GWAS-based PRS and BMI trajectory*

The effect of the interaction between PRS and BMI trajectory was evaluated by including the multiplicative interaction term in the Cox regression model, with additional adjustments for age, sex, race, family history of lung cancer, education level, smoking status, personal history of diabetes, current marital status, study center, and racial background composition using the first 10 principal components from EIGENSTRAT to account for the population substructure.

*One-sample Mendelian randomization*

Genetic instruments for BMI trajectory were identified using the ordinal regression model, with adjustment for age, sex, race, Family history of lung cancer, Education, Smoking status, Personal history of diabetes, Current marital status, Study center, BMI trajectory, and 10 principal components, to clarify the causal role of BMI trajectory on risk of NSCLC. Finally, 12 SNPs were associated with BMI trajectory (P < 5.0×10^-6^) and the effect was estimated using two-stage least squares (2SLS) method from our PLCO sample.

*GWIA*

We then applied the PLINK clumping function (parameters: --clump-kb 1000 --clump-p1 1.00 --clump-p2 1.00 --clump-r2 0.50) to determine the top SNPs. Next, the bootstrap percentile method and permutation test were the testing step. We evaluated whether the SNP×BMI trajectories interaction term was still significant by calculating the 95% confidence interval (CI) (i.e., 2.5% and 97.5% percentiles) based on 10,000 bootstrap samples and the permutation P value. The P value for the permutation test was estimated using the following formula: $P=Pr(|Z_{i}|\geq|Z_{0}|)$, where i is the number of permutation replicates (i.e., 10,000), Zi is the Z statistic of the interaction term for the i-th permutation replicate, and Z0 is the Z statistic of the interaction term for the original data. We also performed an analysis stratified by SNP genotype to examine the modified association of BMI trajectories with NSCLC risk among the specific genotypes. In addition, we further evaluated the interaction term by performing several sensitivity analyses (e.g., additional adjustment for occupation and family history of any cancer).

*Statistical analysis*

We performed stratified analyses to assess the effects of multiple covariates on the NSCLC risk. The *I²* statistic and *P* values for the *χ²* test from *Cochran’s Q* test were used to investigate the heterogeneity among the different stratifications. We further performed sensitivity analyses with the primary model. Model 1-3 did not include the individuals diagnosed with NSCLC at the first 1, 2, or 4 years of follow-up; Model 4 was additionally adjusted for occupation, and family history of any cancer; Model 5 was additionally adjusted for emphysema status, and bronchitis status; Model 6 was additionally adjusted for drinking status; Model 7 excluded the former smokers; Model 8 was additionally adjusted for age at first smoking, smoking intensity, and smoking frequency; Model 9 was additionally adjusted for arm; Model 10 excluded non-European participants. *P* values (two-sided) < 0.05 were deemed significant, and all analyses were performed using R 3.5.3 and PLINK 1.90 software.

# **Table S1. Characteristics of the study subjects for GWAS.**

| **Variable** | **Total (N=13,365)** | **NSCLC (N=1,146)** | **Non-NSCLC (N=12,219)** | **HR(95%CI)** | ***P*-value^*^** |
| --- | --- | --- | --- | --- | --- |
| Age at trial entry (years) |  |  |  |  |  |
| Mean ± SD | 63.50±5.15 | 64.33±5.02 | 63.42±5.16 | 1.04 (1.02,1.05) | 1.77×10^-9^ |
| Sex, N (%) |  |  |  |  |  |
| Male | 10492 (78.50) | 711 (62.04) | 9781 (80.05) | *Reference* |  |
| Female | 2873 (21.50) | 435 (37.96) | 2438 (19.95) | 2.49 (2.21,2.80) | <2×10^-16^ |
| Race, N(%) |  |  |  |  |  |
| White, non-Hispanic | 12202 (91.30) | 1074 (93.72) | 11128 (91.07) | *Reference* |  |
| Black, non-Hispanic | 1065 (7.97) | 72 (6.28) | 993 (8.13) | 0.81 (0.64,1.02) | 0.077 |
| Hispanic | 26 (0.19) | 0 | 26 (0.21) | - | - |
| Asian | 62 (0.46) | 0 | 62 (0.51) | - | - |
| Other | 10 (0.08) | 0 | 10 (0.08) | - | - |
| Family history of lung cancer, N(%) |  |  |  |  |  |
| Absent | 11395 (88.99) | 881 (81.65) | 10514 (89.66) | *Reference* |  |
| Present | 1410 (11.01) | 198 (18.35) | 1212 (10.34) | 1.91 (1.64,2.23) | <2×10^-16^ |
| Education, N(%) |  |  |  |  |  |
| HS or less | 3792 (28.37) | 421 (36.74) | 3371 (27.59) | *Reference* |  |
| Post HS or some college | 4518 (33.80) | 429 (37.43) | 4089 (33.46) | 0.84 (0.73,0.96) | 0.009 |
| College graduate or degree | 5038 (37.70) | 296 (25.83) | 4742 (38.81) | 0.50 (0.43,0.57) | <2×10^-16^ |
| Missing | 17 (0.13) | 0 | 17 (0.14) |  |  |
| BMI at age 20 years, Mean ± SD, kg/m^2^ | 22.60±2.97 | 22.16±2.98 | 22.64±2.97 | 0.94 (0.93,0.96) | 7.35×10^-8^ |
| BMI at age 50 years, Mean ± SD, kg/m^2^ | 26.19±3.80 | 25.27±3.69 | 26.28±3.80 | 0.93 (0.91,0.94) | <2×10^-16^ |
| BMI at baseline, Mean ± SD, kg/m^2^ | 27.48±4.37 | 26.75±4.37 | 27.55±4.36 | 0.96 (0.94,0.97) | 1.64×10^-8^ |
| Smoking status, N(%) |  |  |  |  |  |
| Never | 4949 (37.04) | 107 (9.34) | 4842 (39.63) | *Reference* |  |
| Former | 6473 (48.44) | 627 (54.71) | 5846 (47.85) | 4.67 (3.80,5.73) | <2×10^-16^ |
| Current | 1940 (14.52) | 412 (35.95) | 1528 (12.50) | 11.40 (9.22, 14.10) | <2×10^-16^ |
| Missing | 3 (0.02) | 0 | 3 (0.02) |  |  |
| Personal history of diabetes, N(%) |  |  |  |  |  |
| Absent | 12314 (92.14) | 1050 (91.62) | 11264 (92.18) | *Reference* |  |
| Present | 994 (7.44) | 86 (7.51) | 908 (7.44) | 1.08 (0.86,1.34) | 0.519 |
| Missing | 57 (0.42) | 10 (0.87) | 47 (0.38) |  |  |
| Current marital status, N(%) |  |  |  |  |  |
| Married or living with someone | 10862 (81.27) | 830 (72.43) | 10032 (82.10) | Reference |  |
| Divorced, separated, or widowed | 2080 (15.56) | 290 (25.31) | 1790 (14.65) | 1.99 (1.74,2.27) | <2×10^-16^ |
| Single, never married | 405 (3.03) | 25 (2.18) | 380 (3.11) | 0.84 (0.57,1.25) | 0.394 |
| Missing | 18 (0.14) | 1 (0.08) | 17 (0.14) |  |  |
| Hormone replacement therapy(in female), N(%) |  |  |  |  |  |
| Never | 979 (34.08) | 162 (37.24) | 817 (33.51) | *Reference* |  |
| Current | 1365 (47.51) | 196 (45.06) | 1169 (47.95) | 0.85 (0.69,1.04) | 0.113 |
| Former | 509 (17.72) | 75 (17.24) | 434 (17.80) | 0.86 (0.66,1.14) | 0.293 |
| Unknown or missing | 20 (0.69) | 2 (0.46) | 18 (0.74) |  |  |
| Study center, N(%) |  |  |  |  |  |
| 1= University of Colorado | 1175 (8.79) | 77 (6.72) | 1098 (8.99) | *Reference* |  |
| 2= Georgetown University | 805 (6.02) | 52 (4.54) | 753 (6.16) | 0.97 (0.68,1.37) | 0.845 |
| 3= Pacific Health Research and Education Institute (Honolulu) | 60 (0.45) | 0 | 60 (0.49) | - | - |
| 4= Henry Ford Health System | 1681 (12.58) | 169 (14.75) | 1512 (12.37) | 1.66 (1.27,2.18) | 2.25×10^-4^ |
| 5= University of Minnesota | 3280 (24.54) | 280 (24.43) | 3000 (24.55) | 1.31 (1.01,1.68) | 0.038 |
| 6= Washington University in St Louis | 1220 (9.13) | 129 (11.26) | 1091 (8.93) | 1.72 (1.30,2.28) | 1.73×10^-4^ |
| 8= University of Pittsburgh | 1601 (11.98) | 152 (13.26) | 1449 (11.86) | 1.51 (1.15,1.98) | 0.003 |
| 9= University of Utah | 1213 (9.08) | 77 (6.72) | 1136 (9.30) | 0.97 (0.71,1.33) | 0.865 |
| 10= Marshfield Clinic Research Foundation | 1793 (13.42) | 163 (14.22) | 1630 (13.33) | 1.41 (1.08,1.85) | 0.013 |
| 11= University of Alabama at Birmingham | 537 (4.01) | 47 (4.10) | 490 (4.02) | 1.71 (1.19,2.46) | 0.004 |

^*^ Univariate Cox proportional hazards regression model

GWAS, genome-wide association study; NSCLC, non-small cell lung cancer; HR, hazard ratio; CI, confidence interval

# **Table S2. Summary of the 19 GWAS-identified SNPs associated with lung cancer.**

| **SNP** | **Chr** | **Position ^a^** | **Nearby gene** | **Allele ^b^** | **PMID** | **Previously published study ^c^** | | |  | **PLCO study ^e^** | | |
| --- | --- | --- | --- | --- | --- | --- | --- | --- | --- | --- | --- | --- |
|  |  |  |  |  |  | **OR (95%CI) *** | ***P*-value** | **Population** |  | **EAF ^d^** | **HR (95%CI)** | ***P*-value** |
| rs71658797 | 1 | 77967507 | *AK5* | A/T | 28604730 | 1.10 (1.09,1.18) | 3.30×10^-11^ | European |  | 0.10/0.10 | 1.01 (0.87,1.17) | 0.881 |
| rs31489 | 5 | 1342714 | *CLPTM1L* | A/C | 19836008 | 0.89 (0.86,0.92) | 1.56×10^-10^ | European |  | 0.40/0.42 | 0.92 (0.84,1.00) | 0.055 |
| rs4635969 | 5 | 1308552 | *TERT* | T/C | 19836008 | 0.88 (0.84,0.92) | 2.15×10^-8^ | European |  | 0.19/0.20 | 0.92 (0.82,1.02) | 0.121 |
| rs4236709 | 8 | 32410110 | *NRG1* | G/A | 28604730 | 1.10 (1.09,1.18) | 1.30×10^-10^ | European |  | 0.24/0.22 | 1.12 (1.01,1.24) | 0.026 |
| rs885518 | 9 | 21830157 | *CDNK2A* | G/A | 28604730 | 1.20 (1.11,1.23) | 9.96×10^-10^ | European |  | 0.13/0.11 | 1.17 (1.03,1.33) | 0.018 |
| rs55768116 | 11 | 118108331 | *MPZL3* | C/A | 28604730 | 1.10 (1.07,1.13) | 2.23×10^-13^ | European |  | 0.45/0.47 | 1.11 (1.02,1.21) | 0.021 |
| rs6489769 | 12 | 1072965 | *RAD52* | G/A | 22585858 | 0.83 (0.78,0.89) | 2.30×10^-10^ | European |  | 0.36/0.37 | 0.96 (0.88,1.05) | 0.367 |
| rs55781567 | 15 | 78857986 | *CHRNA5* | G/C | 28604730 | 1.27 (1.23,1.31) | 3.10×10^-103^ | European |  | 0.38/0.33 | 1.20 (1.10,1.32) | 4.87×10^-5^ |
| rs680244 | 15 | 78871288 | *CHRNA5* | T/C | 28604730 | 0.92 (0.90,0.94) | 3.51×10^-13^ | European |  | 0.41/0.43 | 0.91 (0.83,0.99) | 0.027 |
| rs77468143 | 15 | 49376624 | *SECISBP2L* | G/T | 28604730 | 0.86 (0.83,0.89) | 1.70×10^-16^ | European |  | 0.21/0.23 | 0.90 (0.81,1.00) | 0.061 |
| rs8042374 | 15 | 78908032 | *CHRNA3* | A/G | 28604730 | 0.79 (0.77,0.82) | 2.91×10^-60^ | European |  | 0.22/0.24 | 0.90 (0.81,0.99) | 0.039 |
| rs10937405 | 3 | 189383183 | *TP63* | T/C | 20871597 | 1.31 (1.22,1.42) | 7.26×10^-12^ | Asian |  | 0.43/0.42 | 1.03 (0.94,1.12) | 0.566 |
| rs72658409 | 9 | 22160087 | *CDKN2B* | T/C | 26732429 | 0.77 (0.72,0.84) | 1.41×10^-10^ | Asian |  | 0.07/0.08 | 0.84 (0.71,0.99) | 0.040 |
| rs1663689 | 10 | 9025195 | *GATA3* | G/A | 22797725 | 0.88 (0.84,0.91) | 2.84×10^-10^ | Asian |  | 0.22/0.22 | 0.94 (0.85,1.04) | 0.240 |
| rs11610143 | 12 | 52349071 | *ACVR1B* | G/C | 26732429 | 0.89 (0.85,0.92) | 4.96×10^-9^ | Asian |  | 0.20/0.19 | 1.02 (0.91,1.14) | 0.739 |
| rs12296850 | 12 | 100820085 | *NR1H4* | G/A | 23341777 | 0.78 (0.72,0.84) | 1.19×10^-10^ | Asian |  | 0.06/0.07 | 0.90 (0.75,1.08) | 0.247 |
| rs7216064 | 17 | 65898809 | *BPTF* | A/G | 22797724 | 1.20 (1.13,1.26) | 7.40×10^-11^ | Asian |  | 0.20/0.20 | 1.01 (0.91,1.13) | 0.813 |
| rs17728461 | 22 | 30598552 | *MTMR3* | G/C | 21725308 | 1.20 (1.14,1.27) | 1.10×10^-11^ | Asian |  | 0.32/0.30 | 1.04 (0.95,1.14) | 0.378 |
| rs36600 | 22 | 30337586 | *MTMR3* | A/G | 21725308 | 1.29 (1.20,1.38) | 6.20×10^-13^ | Asian |  | 0.26/0.25 | 1.06 (0.96,1.17) | 0.219 |

^a^ Based on Build37 (hg19)

^b^ Effect/other allele

^c^ Obtained from published studies

^d^ Effect allele frequency, individuals with or without NSCLC in PLCO study

^e^ Adjusted for age, sex, race, family history of lung cancer, education, smoking, personal history of diabetes, current marital status, study center, and first10 principal components

* Published OR was transformed into the reverse correspond to the Effect allele

GWAS, genome-wide association study; SNP, single nucleotide polymorphism; Chr, chromosome; EAF, effect allele frequency; OR, odds ratio; HR, hazard ratio; CI, confidence interval; PLCO, Prostate, lung, colorectal, and ovarian

# **Table S3. Association between age-specific BMI and NSCLC risk.**

| **Age-specific BMI** | **NSCLC/Non-NSCLC**  **(2,641/135,469)** | **HR (95%CI)****^a^** | ***P*-value^a^** |
| --- | --- | --- | --- |
| BMI, age 20 years ^a^ |  |  |  |
| <18.5 | 211/10808 | 1.09 (0.94,1.26) | 0.259 |
| 18.5-25 | 2025/103692 | *Reference* |  |
| 25-30 | 364/18527 | 0.87 (0.78,0.98) | 0.025 |
| ≥30 | 41/2442 | 0.72 (0.52,0.99) | 0.047 |
| *Trend* ^b^ |  | 0.94 (0.89,0.99) | 0.014 |
| Continuous, per 5 kg/m^2^ |  | 0.88 (0.82,0.95) | 0.001 |
| BMI, age 50 years old ^a^ |  |  |  |
| <18.5 | 36/980 | 1.79 (1.27,2.52) | 8.28×10^-4^ |
| 18.5-25 | 1358/61586 | *Reference* |  |
| 25-30 | 974/53907 | 0.68 (0.62,0.74) | <2×10^-16^ |
| ≥30 | 273/18996 | 0.54 (0.47,0.63) | <2×10^-16^ |
| *Trend* ^b^ |  | 0.82 (0.79,0.85) | <2×10^-16^ |
| Continuous, per 5 kg/m^2^ |  | 0.70 (0.66,0.74) | <2×10^-16^ |
| BMI at baseline ^a^ |  |  |  |
| <18.5 | 31/897 | 1.77 (1.24,2.53) | 0.002 |
| 18.5-25 | 1008/44133 | *Reference* |  |
| 25-30 | 1102/57857 | 0.71 (0.65,0.77) | 5.15×10^-14^ |
| ≥30 | 500/32582 | 0.55 (0.49,0.62) | <2×10^-16^ |
| *Trend*^b^ |  | 0.83 (0.80,0.86) | <2×10^-16^ |
| Continuous, per 5 kg/m^2^ |  | 0.75 (0.71,0.78) | <2×10^-16^ |

^a^ Cox proportional hazards regression model adjusted for age, sex, race, family history of lung cancer, education, smoking, personal history of diabetes, current marital status, study canter

^b^ Test for linear trend across categories was performed by modeling the levels of BMI categories as a continuous variable in a separate model

BMI, Body mass index; NSCLC, non-small cell lung cancer; HR, hazard ratio; CI, confidence interval

# **Table S4. Sensitivity analysis for the association between BMI trajectory and NSCLC risk.**

| **Model** | **Evaluation method** | **BMI trajectory** | **HR (95%CI) ^a^** | ***P*-value ^a^** |
| --- | --- | --- | --- | --- |
| 1 | Exclusion of incident cancers within the first 1 year | Normal to normal | *Reference* |  |
|  |  | Normal to overweight | 0.72 (0.66,0.79) | 2.84×10^-12^ |
|  |  | Normal to obese | 0.51 (0.45,0.59) | < 2×10^-16^ |
|  |  | Overweight to obese | 0.39 (0.28,0.54) | 2.66×10^-8^ |
|  |  | *Trend* ^b^ | 0.72 (0.68,0.77) | < 2×10^-16^ |
| 2 | Exclusion of incident cancers within the first 2 years | Normal to normal | *Reference* |  |
|  |  | Normal to overweight | 0.86 (0.78,0.95) | 0.003 |
|  |  | Normal to obese | 0.74 (0.64,0.86) | 8.77×10^-5^ |
|  |  | Overweight to obese | 0.62 (0.44,0.88) | 0.007 |
|  |  | *Trend* ^b^ | 0.86 (0.81,0.92) | 2.53×10^-6^ |
| 3 | Exclusion of incident cancers within the first 4 years | Normal to normal | *Reference* |  |
|  |  | Normal to overweight | 0.87 (0.78,0.97) | 0.011 |
|  |  | Normal to obese | 0.78 (0.66,0.91) | 0.002 |
|  |  | Overweight to obese | 0.60 (0.40,0.90) | 0.012 |
|  |  | *Trend* ^b^ | 0.87 (0.81,0.93) | 9.66×10^-5^ |
| 4 | Additional adjustment for occupation and family history of any cancer | Normal to normal | *Reference* |  |
|  |  | Normal to overweight | 0.77 (0.71,0.84) | 1.12×10^-8^ |
|  |  | Normal to obese | 0.60 (0.52,0.69) | 4.59×10^-13^ |
|  |  | Overweight to obese | 0.53 (0.39,0.73) | 6.84×10^-5^ |
|  |  | *Trend* ^b^ | 0.78 (0.74,0.83) | < 2×10^-16^ |
| 5 | Additional adjustment for emphysema or bronchitis | Normal to normal | *Reference* |  |
|  |  | Normal to overweight | 0.79 (0.72,0.86) | 2.02×10^-7^ |
|  |  | Normal to obese | 0.62 (0.54,0.71) | 8.23×10^-12^ |
|  |  | Overweight to obese | 0.56 (0.41,0.76) | 2.23×10^-4^ |
|  |  | *Trend* ^b^ | 0.80 (0.75,0.84) | 1.13×10^-14^ |
| 6 | Additional adjustment for drinking status | Normal to normal | *Reference* |  |
|  |  | Normal to overweight | 0.75 (0.68, 0.84) | 4.81×10^-7^ |
|  |  | Normal to obese | 0.65 (0.55, 0.76) | 3.47×10^-7^ |
|  |  | Overweight to obese | 0.51 (0.35, 0.76) | 7.97×10^-4^ |
|  |  | *Trend* ^b^ | 0.79 (0.74, 0.85) | 2.50×10^-10^ |
| 7 | Exclusion of participants that were former smokers | Normal to normal | *Reference* |  |
|  |  | Normal to overweight | 0.80 (0.68, 0.93) | 0.005 |
|  |  | Normal to obese | 0.67 (0.52, 0.87) | 0.003 |
|  |  | Overweight to obese | 0.51 (0.28, 0.94) | 0.032 |
|  |  | *Trend* ^b^ | 0.81 (0.73, 0.90) | 9.36×10^-5^ |
| 8 | Additional adjustment for age at first smoking, smoking intensity, and smoking frequency | Normal to normal | *Reference* |  |
|  |  | Normal to overweight | 0.83 (0.75,0.91) | 6.85×10^-5^ |
|  |  | Normal to obese | 0.68 (0.59,0.79) | 2.02×10^-7^ |
|  |  | Overweight to obese | 0.57 (0.41,0.80) | 0.001 |
|  |  | *Trend* ^b^ | 0.83 (0.78,0.88) | 1.19×10^-9^ |
| 9 | Additional adjustment for the arm | Normal to normal | *Reference* |  |
|  |  | Normal to overweight | 0.86 (0.79,0.94) | 7.49×10^-4^ |
|  |  | Normal to obese | 0.73 (0.63,0.83) | 5.58×10^-6^ |
|  |  | Overweight to obese | 0.66 (0.48,0.90) | 0.009 |
|  |  | *Trend* ^b^ | 0.86 (0.81,0.91) | 2.16×10^-7^ |
| 10 | Exclusion of participants that were non-European ancestry | Normal to normal | *Reference* |  |
|  |  | Normal to overweight | 0.86 (0.78,0.94) | 0.001 |
|  |  | Normal to obese | 0.73 (0.63,0.84) | 1.78×10^-5^ |
|  |  | Overweight to obese | 0.62 (0.44,0.88) | 0.007 |
|  |  | *Trend* ^b^ | 0.85 (0.80,0.91) | 4.93×10^-7^ |

^a^ Cox proportional hazards regression model adjusted for age, sex, race, family history of lung cancer, education, smoking, personal history of diabetes, current marital status, study canter

^b^ Test for linear trend across categories was performed by modelling the levels of BMI categories as a continuous variable in a separate model

BMI, Body mass index; NSCLC, non-small cell lung cancer; HR, hazard ratio; CI, confidence interval

# **Table S5. Association of *s*PRS_GWAS_ and *w*PRS_GWAS_ with NSCLC risk.**

| **Poly-geneticriskscore** | **NSCLC/Non-NSCLC**  **(1,146/12,219)** | **HR (95%CI) ^a^** | ***P*-value ^a^** |
| --- | --- | --- | --- |
| ***s*PRS_GWAS_** |  |  |  |
| Low (<24) | 325/4382 | *Reference* |  |
| Intermediate (25-26) | 231/2422 | 1.33 (1.12,1.59) | 0.001 |
| High (≥27) | 376/3183 | 1.56 (1.34,1.82) | 1.62×10^-8^ |
| *Trend*^b^ |  | 1.25 (1.16,1.35) | 1.44×10^-8^ |
| ***w*PRS_GWAS_** |  |  |  |
| Low (<3.971) | 242/3408 | *Reference* |  |
| Intermediate (3.971-4.480) | 318/3323 | 1.37 (1.16,1.63) | 3.95×10^-4^ |
| High (≥4.480) | 372/3256 | 1.52 (1.29,1.81) | 1.19×10^-6^ |
| *Trend*^b^ |  | 1.23 (1.13,1.34) | 1.55×10^-6^ |

^a^ Cox proportional hazards regression model adjusted for age, sex, race, family history of lung cancer, education, smoking, personal history of diabetes, current marital status, study center, BMI at baseline, and first10 principal components

^b^ Test for linear trend across categories was performed by modelling the levels of PRS as a continuous variable in a separate model

NSCLC, non-small cell lung cancer; HR, hazard ratio; HR, hazard ratio; CI, confidence interval

# **Table S6. Interaction analysis between age-specific BMI and the *s*PRS_GWAS_.**

| **Age-specific BMI** | **Low**^a^ | | **Intermediate**^a^ | | **High**^a^ | | ***P_interaction_^d^*** |
| --- | --- | --- | --- | --- | --- | --- | --- |
|  | **HR (95%CI)**^b^ | ***P-*value**^b^ | **HR (95%CI)**^b^ | ***P-*value**^b^ | **HR (95%CI)**^b^ | ***P-*value**^b^ |  |
| BMI, age 20 years |  |  |  |  |  |  |  |
| <18.5 | 1.11 (0.74, 1.64) | 0.618 | 1.28 (0.79, 2.07) | 0.320 | 1.11 (0.74, 1.68) | 0.605 | 0.471 |
| 18.5-25 | *Reference* |  | *Reference* |  | *Reference* |  |  |
| 25-30 | 0.63 (0.42, 0.93) | 0.022 | 0.65 (0.42, 1.00) | 0.052 | 1.07 (0.80, 1.44) | 0.648 |  |
| ≥30 | 1.08 (0.51, 2.32) | 0.835 | 0.64 (0.16, 2.61) | 0.536 | 0.38 (0.12, 1.20) | 0.100 |  |
| *Trend*^c^ | 0.88 (0.76, 1.03) | 0.110 | 0.85 (0.71, 1.03) | 0.093 | 0.98 (0.86, 1.12) | 0.761 |  |
| BMI, age 50 years |  |  |  |  |  |  |  |
| <18.5 | 4.35 (1.99, 9.50) | 2.25×10^-4^ | 1.12(0.15, 7.59) | 0.914 | 2.44 (0.96, 6.20) | 0.062 | 0.843 |
| 18.5-25 | *Reference* |  | *Reference* |  | *Reference* |  |  |
| 25-30 | 0.67 (0.52, 0.86) | 0.002 | 0.76 (0.52, 0.95) | 0.022 | 0.76 (0.60, 0.96) | 0.022 |  |
| ≥30 | 0.69 (0.47, 1.02) | 0.060 | 0.64 (0.41, 1.00) | 0.050 | 0.51 (0.34, 0.76) | 0.001 |  |
| *Trend*^c^ | 0.85 (0.77, 0.94) | 0.002 | 0.85 (0.76, 0.96) | 0.009 | 0.84 (0.76, 0.93) | 5.02×10^-4^ |  |
| BMI, age at baseline |  |  |  |  |  |  |  |
| <18.5 | 1.05 (0.26, 4.29) | 0.948 | 0.81 (0.19, 3.40) | 0.778 | 2.84 (0.88, 9.17) | 0.082 | 0.702 |
| 18.5-25 | *Reference* |  | *Reference* |  | *Reference* |  |  |
| 25-30 | 0.64 (0.49, 0.83) | 7.39×10^-4^ | 0.85 (0.62, 1.15) | 0.290 | 0.78 (0.61, 0.99) | 0.044 |  |
| ≥30 | 0.64 (0.46, 0.87) | 0.005 | 0.58 (0.38, 0.87) | 0.009 | 0.62 (0.46, 0.84) | 0.002 |  |
| *Trend*^c^ | 0.84 (0.76, 0.93) | 5.53×10^-4^ | 0.87 (0.77, 0.97) | 0.017 | 0.86 (0.78, 0.94) | 0.001 |  |

^a^ Low < 24; Intermediate, 25~26; High > 27

^b^ Cox proportional hazards regression model adjusted for age, sex, race, family history of lung cancer, education, smoking, personal history of diabetes, current marital status, study center, and first 10 principal components

^c^ Test for linear trend across categories was performed by modelling the levels of BMI categories as a continuous variable in a separate model

^d^ *P* value was obtained from the interaction term between GWAS-based PRS and age-specific BMI

BMI, Body mass index; HR, hazard ratio; CI, confidence interval

# **Table S7. Interaction analysis between age-specific BMI and the *w*PRS_GWAS_.**

| **Age-specific BMI** | **Low** | **Intermediate** | | | | **High** | | ***P_interaction_^c^*** |
| --- | --- | --- | --- | --- | --- | --- | --- | --- |
|  | **HR (95%CI)** ^a^ | ***P-*value** ^a^ | **HR (95%CI)** ^a^ | ***P*-value** ^a^ | **HR (95%CI)** ^a^ | | ***P-*value** ^a^ |  |
| BMI, age 20 years |  |  |  |  |  | |  |  |
| <18.5 | 0.97 (0.61,1.53) | 0.880 | 1.33 (0.88,2.03) | 0.178 | 1.14 (0.76,1.71) | | 0.515 | 0.969 |
| 18.5-25 | *Reference* |  | *Reference* |  | *Reference* | |  |  |
| 25-30 | 0.54 (0.33,0.88) | 0.013 | 0.73 (0.51,1.03) | 0.073 | 1.06 (0.78,1.44) | | 0.705 |  |
| ≥30 | 1.38 (0.64,2.98) | 0.409 | 0.32 (0.08,1.29) | 0.108 | 0.47 (0.15,1.47) | | 0.195 |  |
| *Trend*^b^ | 0.86 (0.72,1.03) | 0.113 | 0.86 (0.74,0.99) | 0.049 | 0.99 (0.87,1.14) | | 0.911 |  |
| BMI, age 50 years |  |  |  |  |  | |  |  |
| <18.5 | 4.57 (1.80,11.62) | 0.001 | 2.57 (0.92,7.17) | 0.071 | 1.95 (0.69,5.47) | | 0.205 | 0.661 |
| 18.5-25 | *Reference* |  | *Reference* |  | *Reference* | |  |  |
| 25-30 | 0.77 (0.57,1.03) | 0.077 | 0.61 (0.47,0.79) | 1.76×10^-4^ | 0.75 (0.59,0.95) | | 0.017 |  |
| ≥30 | 0.84 (0.54,1.30) | 0.433 | 0.55 (0.37,0.81) | 0.002 | 0.53 (0.35,0.79) | | 0.002 |  |
| *Trend*^b^ | 0.91 (0.80,1.02) | 0.105 | 0.80 (0.72,0.89) | 2.79×10^-5^ | 0.84 (0.76,0.93) | | 6.69×10^-4^ |  |
| BMI, age at baseline |  |  |  |  |  | |  |  |
| <18.5 | 1.16 (0.28,4.82) | 0.834 | 0.92 (0.22,3.80) | 0.907 | 1.98 (0.61,6.41) | | 0.256 | 0.689 |
| 18.5-25 | *Reference* |  | *Reference* |  | *Reference* | |  |  |
| 25-30 | 0.62 (0.46,0.84) | 0.002 | 0.84 (0.65,1.08) | 0.175 | 0.73 (0.57,0.94) | | 0.013 |  |
| ≥30 | 0.65 (0.45,0.93) | 0.018 | 0.54 (0.38,0.77) | 5.85×10^-4^ | 0.65 (0.48,0.87) | | 0.005 |  |
| *Trend*^b^ | 0.84 (0.75,0.94) | 0.003 | 0.85 (0.77,0.94) | 0.002 | 0.86 (0.78,0.94) | | 0.002 |  |

^a^ Cox proportional hazards regression model adjusted for age, sex, race, family history of lung cancer, education, smoking, personal history of diabetes, current marital status, study center, and first10 principal components

^b^ Test for linear trend across categories was performed by modelling the levels of BMI categories as a continuous variable in a separate model

^c^ *P* value was obtained from the interaction term between GWAS-based PRS and age-specific BMI

BMI, Body mass index; HR, hazard ratio; CI, confidence interval

# **Table S8. Summary of four independent SNPs identified by GWIA.**

| **Locus** | **SNP** | **Position ^a^** | **Nearby gene** | **Allele^b^** | **EAF^c^** | **HR_interaction_ (95%CI)^d^** | ***P*_interaction_** | **95%CI^e^** | ***P*^f^** |
| --- | --- | --- | --- | --- | --- | --- | --- | --- | --- |
| 12q14.1 | rs79297227 | 60057271 | SLC16A7 | C/T | 0.05/0.05 | 1.90 (1.50, 2.50) | 1.01×10^-7^ | 1.53-2.69 | <0.001 |
| 3p22.3 | rs2336652 | 34519341 | *CLASP2* | A/C | 0.07/0.06 | 1.70 (1.40, 2.10) | 3.92×10^-7^ | 1.36-2.24 | <0.001 |
| 19p13.2 | rs16018 | 13411482 | *CACNA1A* | G/A | 0.31/0.31 | 1.40 (1.20, 1.60) | 3.92×10^-7^ | 1.25-1.77 | <0.001 |
| 7q34 | rs4726760 | 139998886 | *BRAF* | T/C | 0.12/0.13 | 0.62 (0.50, 0.75) | 9.19×10^-7^ | 0.47-0.74 | <0.001 |

^a^ Based on Build37 (hg19)

^b^ Effect/otherallele

^c^ Effect allele frequency, individuals with or without NSCLC in PLCO study

^d^ Estimated by Cox regression model with the adjustment for age, sex, race, family history of lung cancer, education, smoking status, personal history of diabetes, current marital status, study center, and first 10 principal components

^e^ Estimated by bootstrap percentile method based on 10,000 bootstrap samples

^f^ Estimated by permutation test with 10,000 permutations

SNPs, single nucleotide polymorphisms; GWIA, Genome-wide interaction analysis; HR, hazard ratio; CI, confidence interval

# **Table S9. Sensitivity analyses** **for the** **interaction between BMI trajectory and** **rs79297227, rs2336652, rs16018 and rs79297227.**

| **Model** | **Evaluation method** | **SNP** | **HR_interaction_ (95%CI) ^a^** | ***P*_interaction_ ^a^** |
| --- | --- | --- | --- | --- |
| 1 | Exclusion of incident cancers within the first 1 year | rs79297227 | 2.00 (1.55,2.58) | 8.20×10^-8^ |
|  |  | rs2336652 | 1.68 (1.36,2.08) | 1.84×10^-6^ |
|  |  | rs16018 | 1.37 (1.20,1.55) | 1.91×10^-6^ |
|  |  | rs4726760 | 0.65 (0.53,0.80) | 4.79×10^-5^ |
| 2 | Exclusion of incident cancers within the first 2 years | rs79297227 | 1.85 (1.42,2.41) | 5.62×10^-6^ |
|  |  | rs2336652 | 1.68 (1.35,2.09) | 3.02×10^-6^ |
|  |  | rs16018 | 1.37 (1.20,1.57) | 2.14×10^-6^ |
|  |  | rs4726760 | 0.64 (0.52,0.79) | 3.98×10^-5^ |
| 3 | Exclusion of incident cancers within the first 4 years | rs79297227 | 1.86 (1.38,2.50) | 3.93×10^-5^ |
|  |  | rs2336652 | 1.66 (1.31,2.11) | 2.32×10^-5^ |
|  |  | rs16018 | 1.41 (1.23,1.63) | 1.85×10^-6^ |
|  |  | rs4726760 | 0.64 (0.51,0.81) | 1.97×10^-4^ |
| 4 | Additional adjustment for occupation and family history of any cancer | rs79297227 | 2.01 (1.56, 2.61) | 1.15×10^-7^ |
|  |  | rs2336652 | 1.72 (1.35, 2.20) | 1.36×10^-5^ |
|  |  | rs16018 | 1.47 (1.24, 1.74) | 8.59×10^-6^ |
|  |  | rs4726760 | 0.59 (0.47, 0.74) | 2.98×10^-6^ |
| 5 | Additional adjustment for emphysema or bronchitis | rs79297227 | 1.91 (1.45, 2.47) | 9.38×10^-7^ |
|  |  | rs2336652 | 1.71 (1.34, 2.18) | 1.64×10^-5^ |
|  |  | rs16018 | 1.49 (1.26, 1.76) | 3.98×10^-6^ |
|  |  | rs4726760 | 0.58 (0.46, 0.72) | 1.29×10^-6^ |
| 6 | Additional adjustment for drinking status | rs79297227 | 1.91 (1.44, 2.54) | 7.11×10^-6^ |
|  |  | rs2336652 | 1.76 (1.34, 2.31) | 4.53×10^-5^ |
|  |  | rs16018 | 1.54 (1.27, 1.86) | 1.16×10^-5^ |
|  |  | rs4726760 | 0.61 (0.48, 0.79) | 1.41×10^-4^ |
| 7 | Exclusion of participants that were former smokers | rs79297227 | 2.61 (1.69, 4.05) | 1.66×10^-5^ |
|  |  | rs2336652 | 1.54 (1.05, 2.25) | 0.026 |
|  |  | rs16018 | 1.49 (1.12, 1.99) | 0.007 |
|  |  | rs4726760 | 0.54 (0.37, 0.77) | 6.80×10^-4^ |
| 8 | Additional adjustment for age at first smoking, smoking intensity, and smoking frequency | rs79297227 | 1.86 (1.42,2.43) | 6.47×10^-6^ |
|  |  | rs2336652 | 1.70 (1.37,2.12) | 1.84×10^-6^ |
|  |  | rs16018 | 1.30 (1.14,1.48) | 9.72×10^-5^ |
|  |  | rs4726760 | 0.64 (0.52,0.79) | 2.63×10^-5^ |
| 9 | Additional adjustment for arm | rs79297227 | 1.95 (1.53,2.49) | 8.89×10^-8^ |
|  |  | rs2336652 | 1.76 (1.43,2.18) | 1.44×10^-7^ |
|  |  | rs16018 | 1.37 (1.21,1.55) | 6.35×10^-7^ |
|  |  | rs4726760 | 0.63 (0.51,0.77) | 5.61×10^-6^ |
| 10 | Exclusion of participants that were non-European ancestry | rs79297227 | 1.93 (1.51,2.48) | 1.73×10^-7^ |
|  |  | rs2336652 | 1.45 (1.13,1.87) | 0.004 |
|  |  | rs16018 | 1.31 (1.15,1.50) | 5.48×10^-5^ |
|  |  | rs4726760 | 0.60 (0.49,0.74) | 1.38×10^-6^ |

^a^ Likelihood ratio test was performed by comparing two Cox regression models, one with and one without an interaction term for a SNP and BMI trajectory

BMI, Body mass index; SNPs, single nucleotide polymorphisms; HR, hazard ratio; CI, confidence interval

# **Table S10. Single-nucleotide polymorphisms were used as instrumental variables in the multivariable Mendelian randomization analyses of BMI trajectory.**

| **Chr** | **SNP** | **Position** | **BMI trajectory association^b^** | | | **EAF^c^** | **Lung cancer association^d^** | |
| --- | --- | --- | --- | --- | --- | --- | --- | --- |
|  |  |  | **Allele^a^** | **Beta** | **SE** |  | **HR (95%CI)** | ***P* value** |
| 2 | rs10188334 | 653874 | T/C | -0.1865 | 0.0349 | 0.17 | 0.92(0.81,1.04) | 0.171 |
| 2 | rs11680644 | 109988215 | C/G | -0.1227 | 0.0260 | 0.45 | 1.03 (0.94,1.13) | 0.474 |
| 2 | rs45567336 | 113935370 | G/A | 0.2032 | 0.0409 | 0.11 | 0.97 (0.84,1.13) | 0.721 |
| 3 | rs62259549 | 82899080 | C/T | 0.4852 | 0.0950 | 0.02 | 1.32 (0.97,1.80) | 0.082 |
| 4 | rs10016061 | 77203117 | C/G | 0.1601 | 0.0349 | 0.17 | 1.02 (0.91,1.16) | 0.692 |
| 7 | rs141478780 | 95931940 | T/C | 0.3255 | 0.0699 | 0.03 | 0.94 (0.73,1.20) | 0.616 |
| 9 | rs718841 | 117198035 | A/T | -0.1348 | 0.0283 | 0.29 | 1.07 (0.97,1.18) | 0.181 |
| 16 | rs11075987 | 53815161 | T/G | -0.1469 | 0.0256 | 0.48 | 0.98 (0.90,1.07) | 0.656 |
| 16 | rs3751813 | 53818708 | G/T | -0.1180 | 0.0258 | 0.45 | 1.01 (0.93,1.11) | 0.749 |
| 16 | rs11075993 | 53837144 | T/G | 0.1864 | 0.0266 | 0.39 | 1.02 (0.93,1.12) | 0.648 |
| 18 | rs2155759 | 24067931 | A/G | 0.1216 | 0.0265 | 0.45 | 0.98 (0.89,1.07) | 0.600 |
| 18 | rs688671 | 57867526 | G/A | 0.1307 | 0.0279 | 0.29 | 1.01 (0.91,1.11) | 0.892 |

^a^ Effect/reference allele

^b^ The beta (β) estimates and standard errors (SE) of BMI trajectory-associated SNPs from the PLCO study

^c^ Effect allele frequent of BMI trajectory-associated SNPs

^d^ Results (HR, 95%CI and *P* value) were derived from PLCO study and were adjusted for age, sex, race, Family history of lung cancer, Education, Smoking status, Personal history of diabetes, Current marital status, Study center, BMI trajectory, 10 principal components

BMI, Body mass index; SNPs, single nucleotide polymorphisms; HR, hazard ratio; CI, confidence interval

# **Table S11. Sensitivity analysis of the relationship between BMI trajectory and lung cancer using one-sample Mendelian randomization.**

| **Mendelian randomization** | **Beta** | **95%CI** | ***P*-value** |
| --- | --- | --- | --- |
| IVW | 0.040 | -0.156,0.235 | 0.690 |
| Egger | 0.561 | -0.079,1.202 | 0.086 |
| (intercept) | -0.087 | -0.189,0.015 | 0.094 |
| Simple median | -0.035 | -0.298,0.227 | 0.792 |

BMI, Body mass index; CI, confidence interval

# **Table S12. Interaction analysis between age-specific BMI/BMI trajectories and the *w*PRS_GWIA_.**

| **Age-specific BMI** | **Low** | **Intermediate** | | | | **High** | | ***P_interaction_^c^*** |
| --- | --- | --- | --- | --- | --- | --- | --- | --- |
|  | **HR (95%CI)** ^a^ | ***P-*value** ^a^ | **HR (95%CI)** ^a^ | ***P*-value** ^a^ | **HR (95%CI)** ^a^ | | ***P-*value** ^a^ |  |
| BMI, age 20 years |  |  |  |  |  | |  |  |
| <18.5 | 1.25 (0.92, 1.69) | 0.155 | 0.98 (0.60, 1.60) | 0.940 | 0.92 (0.55, 1.56) | | 0.766 | 5.06×10^-4^ |
| 18.5-25 | *Reference* |  | *Reference* |  | *Reference* | |  |  |
| 25-30 | 0.64 (0.47, 0.86) | 0.003 | 0.73 (0.51, 1.05) | 0.093 | 1.51 (1.08, 2.12) | | 0.016 |  |
| ≥30 | 0.33 (0.11, 1.04) | 0.059 | 1.32 (0.65, 2.69) | 0.442 | 0.86 (0.32, 2.35) | | 0.775 |  |
| *Trend*^b^ | 0.83 (0.73, 0.94) | 0.003 | 0.92 (0.80, 1.07) | 0.303 | 1.14 (0.98, 1.33) | | 0.081 |  |
| BMI, age 50 years |  |  |  |  |  | |  |  |
| <18.5 | 2.71 (1.27, 5.82) | 0.010 | 5.59 (1.70, 1.83) | 0.005 | 1.32 (0.40, 4.42) | | 0.650 | 2.04×10^-6^ |
| 18.5-25 | *Reference* |  | *Reference* |  | *Reference* | |  |  |
| 25-30 | 0.60 (0.49, 7.32) | 4.56×10^-7^ | 0.89 (0.69, 1.16) | 0.396 | 0.85 (0.63, 1.15) | | 0.287 |  |
| ≥30 | 0.39 (0.27, 0.56) | 2.40×10^-7^ | 0.76 (0.51, 1.13) | 0.176 | 1.34 (0.92, 1.94) | | 0.125 |  |
| *Trend*^b^ | 0.76 (0.70, 0.82) | 7.13×10^-11^ | 0.93 (0.84, 1.04) | 0.184 | 1.03 (0.91, 1.16) | | 0.646 |  |
| BMI, age at baseline |  |  |  |  |  | |  |  |
| <18.5 | 1.75 (0.76, 3.99) | 0.186 | 0.64 (0.09, 4.67) | 0.661 | 1.77 (0.24, 1.31) | | 0.574 | 2.98×10^-9^ |
| 18.5-25 | *Reference* |  | *Reference* |  | *Reference* | |  |  |
| 25-30 | 0.64 (0.52, 0.78) | 9.24×10^-6^ | 0.98 (0.75, 1.29) | 0.900 | 0.98 (0.70, 1.37) | | 0.905 |  |
| ≥30 | 0.39 (0.29, 0.51) | 2.12×10^-11^ | 0.73 (0.52, 1.03) | 0.072 | 1.51 (1.07, 2.13) | | 0.019 |  |
| *Trend*^b^ | 0.76 (0.70, 0.82) | 1.37×10^-12^ | 0.93 (0.84, 1.03) | 0.154 | 1.13 (0.99, 1.27) | | 0.052 |  |
| BMI trajectory |  |  |  |  |  | |  |  |
| Normal to normal | *Reference* |  | *Reference* |  | *Reference* | |  | 3.80×10^-16^ |
| Normal to overweight | 0.57 (0.47, 0.70) | 1.77×10^-8^ | 0.87 (0.67, 1.14) | 0.311 | 1.35 (0.97, 1.88) | | 0.074 |  |
| Normal to obese | 0.31 (0.22, 0.44) | 3.18×10^-11^ | 0.63 (0.42, 0.94) | 0.024 | 1.97 (1.34, 2.91) | | 6.26×10^-4^ |  |
| Overweight to obese | inf | 0.990 | 0.81 (0.39, 1.68) | 0.571 | 1.37 (0.58, 3.24) | | 0.467 |  |
| *Trend*^b^ | 0.54 (0.47, 0.62) | <2×10^-16^ | 0.85 (0.72, 0.99) | 0.043 | 1.30 (1.10, 1.54) | | 0.002 |  |

^a^ Cox proportional hazards regression model adjusted for age, sex, race, family history of lung cancer, education, smoking, personal history of diabetes, current marital status, study center, and first 10 principal components

^b^ Test for linear trend across categories was performed by modelling the levels of BMI categories as a continuous variable in a separate model

^c^ *P* value was obtained from the interaction term between weighted PRS and age-specific BMI/BMI trajectory

BMI, Body mass index; HR, hazard ratio; CI, confidence interval

# **Table S13. Interaction analysis between age-specific BMI/BMI trajectories and the *s*PRS_GWIA_.**

| **Age-specific BMI** | **Low**^a^ | **Intermediate**^a^ | | | | **High**^a^ | | ***P_interaction_^d^*** |
| --- | --- | --- | --- | --- | --- | --- | --- | --- |
|  | **HR (95%CI)**^b^ | ***P-*value**^b^ | **HR (95%CI)**^b^ | ***P*-value**^b^ | **HR (95%CI)**^b^ | | ***P-*value**^b^ |  |
| BMI, age 20 years |  |  |  |  |  | |  |  |
| <18.5 | 1.14 (0.77, 1.69) | 0.517 | 1.23 (0.85, 1.78) | 0.272 | 0.92 (0.58, 1.45) | | 0.714 | 0.321 |
| 18.5-25 | *Reference* |  | *Reference* |  | *Reference* | |  |  |
| 25-30 | 0.76 (0.53, 1.08) | 0.123 | 0.80 (0.58, 1.09) | 0.150 | 0.97 (0.71, 1.34) | | 0.863 |  |
| ≥30 | 0.59 (0.19, 1.86) | 0.370 | 1.09 (0.56, 2.13) | 0.792 | 0.48 (0.15, 1.51) | | 0.211 |  |
| *Trend*^c^ | 0.89 (0.76, 1.04) | 0.137 | 0.95 (0.84, 1.08) | 0.423 | 0.94 (0.82, 1.09) | | 0.428 |  |
| BMI, age 50 years |  |  |  |  |  | |  |  |
| <18.5 | 2.24 (0.81, 6.20) | 0.120 | 6.18 (2.49, 15.33) | 8.64×10^-5^ | 1.59 (0.56, 4.47) | | 0.380 | 0.022 |
| 18.5-25 | *Reference* |  | *Reference* |  | *Reference* | |  |  |
| 25-30 | 0.62 (0.48, 0.80) | 1.96×10^-4^ | 0.85 (0.67, 1.06) | 0.143 | 0.72 (0.56, 0.93) | | 0.010 |  |
| ≥30 | 0.49 (0.33, 0.74) | 7.43×10^-4^ | 0.77 (0.55, 1.10) | 0.148 | 0.78 (0.55, 1.12) | | 0.182 |  |
| *Trend*^c^ | 0.79 (0.71, 0.88) | 7.69×10^-6^ | 0.92 (0.84, 1.01) | 0.076 | 0.88 (0.80, 0.98) | | 0.021 |  |
| BMI, age at baseline |  |  |  |  |  | |  |  |
| <18.5 | 1.87 (0.68, 5.18) | 0.229 | 0.93 (0.23, 3.81) | 0.925 | 2.10 (0.49, 8.94) | | 0.317 | 1.39×10^-4^ |
| 18.5-25 | *Reference* |  | *Reference* |  | *Reference* | |  |  |
| 25-30 | 0.61 (0.48, 0.79) | 1.20×10^-4^ | 0.87 (0.69, 1.10) | 0.259 | 0.88 (0.67, 1.16) | | 0.370 |  |
| ≥30 | 0.40 (0.28, 0.56) | 1.79×10^-7^ | 0.78 (0.59, 1.03) | 0.083 | 0.91 (0.67, 1.24) | | 0.557 |  |
| *Trend*^c^ | 0.75 (0.68, 0.83) | 1.19×10^-8^ | 0.92 (0.85, 1.01) | 0.080 | 0.96 (0.87, 1.06) | | 0.441 |  |
| BMI trajectory |  |  |  |  |  | |  |  |
| Normal to normal | *Reference* |  | *Reference* |  | *Reference* | |  | 6.61×10^-5^ |
| Normal to overweight | 0.61 (0.48, 0.79) | 9.63×10^-5^ | 0.82 (0.65, 1.03) | 0.082 | 0.88 (0.67, 1.14) | | 0.321 |  |
| Normal to obese | 0.40 (0.27, 0.61) | 1.29×10^-5^ | 0.67 (0.48, 0.94) | 0.020 | 0.86 (0.60, 1.22) | | 0.395 |  |
| Overweight to obese | Inf | 0.992 | 0.78 (0.40, 1.55) | 0.487 | 0.52 (0.21, 1.29) | | 0.158 |  |
| *Trend* ^c^ | 0.59 (0.50, 0.70) | 1.87×10^-9^ | 0.84 (0.73, 0.97) | 0.020 | 0.89 (0.76, 1.04) | | 0.149 |  |

^a^ Low = 0; Intermediate =1; High > 2

^b^ Cox proportional hazards regression model adjusted for age, sex, race, family history of lung cancer, education, smoking, personal history of diabetes, current marital status, study center, and first 10 principal components

^c^ Test for linear trend across categories was performed by modelling the levels of BMI categories as a continuous variable in a separate model

^d^ *P* value was obtained from the interaction term between simple PRS and age-specific BMI/BMI trajectory

BMI, Body mass index; HR, hazard ratio; CI, confidence interval


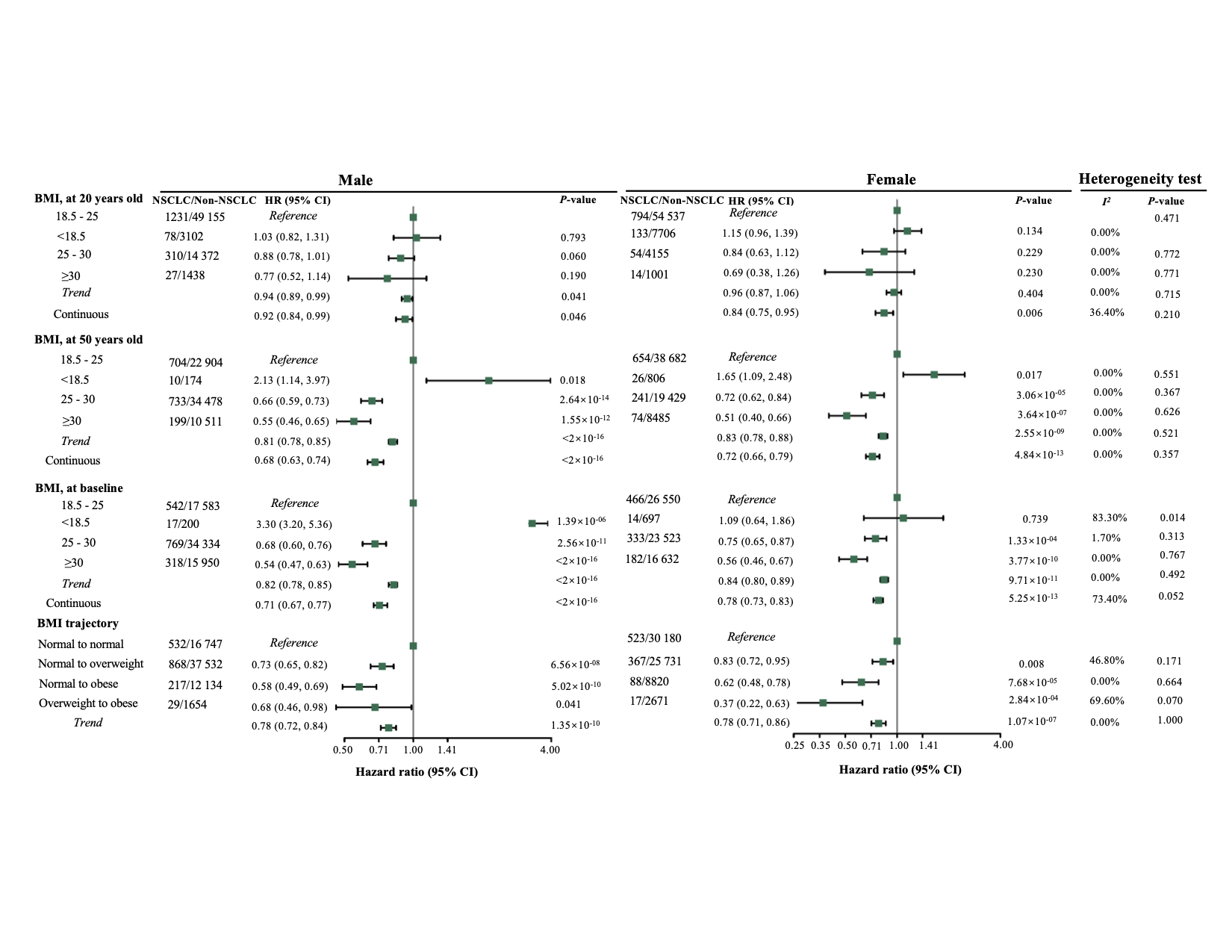


# **Figure S1. Stratification analysis for age-specific BMI and BMI trajectory on NSCLC risk by sex.**

HR and 95% CI were estimated with the adjustment for age, sex, race, family history of lung cancer, education, smoking status, personal history of diabetes, current marital status, study center

BMI, Body mass index; NSCLC, non-small cell lung cancer; HR, hazard ratio; CI, confidence interval

**
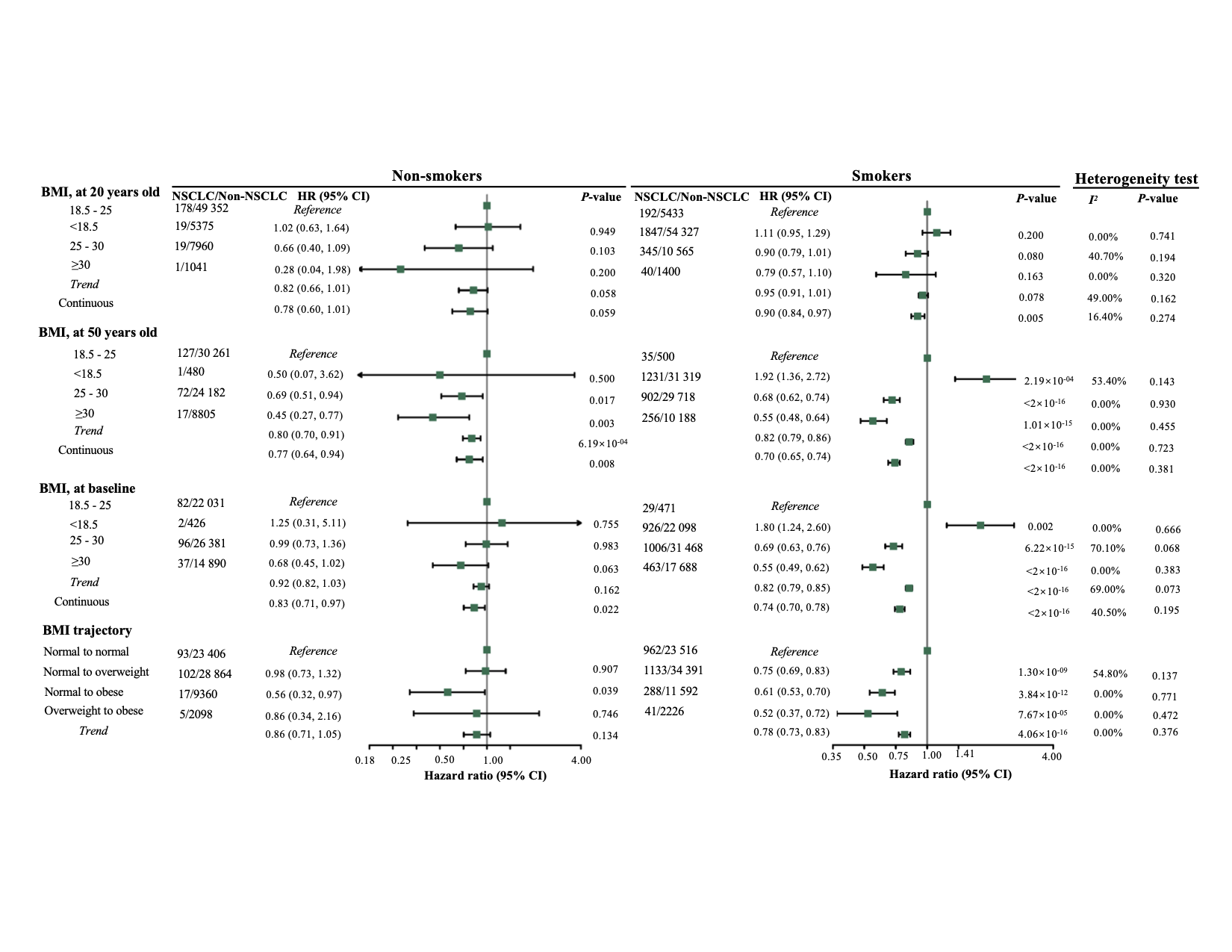
**

# **Figure S2. Stratification analysis for age-specific BMI and BMI trajectory on NSCLC risk by smoking status.**

HR and 95% CI were estimated with the adjustment for age, sex, race, family history of lung cancer, education, smoking status, personal history of diabetes, current marital status, study center

BMI, Body mass index; NSCLC, non-small cell lung cancer; HR, hazard ratio; CI, confidence interval


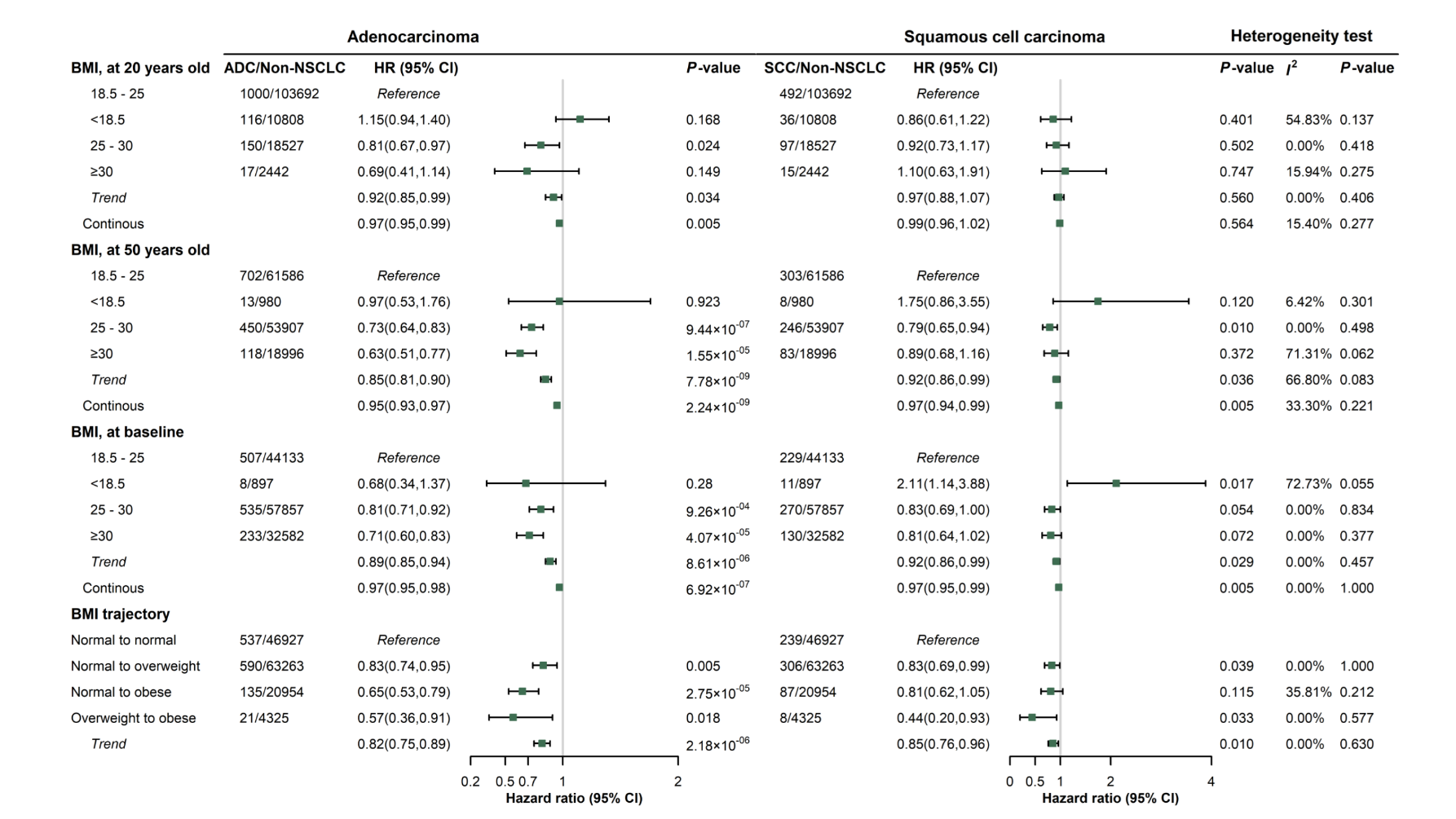


# **Figure S3. Stratification analysis for age-specific BMI and BMI trajectory on NSCLC risk by histological type.**

HR and 95% CI were estimated with the adjustment for age, sex, race, family history of lung cancer, education, smoking status, personal history of diabetes, current marital status, study center

BMI, Body mass index; NSCLC, non-small cell lung cancer; HR, hazard ratio; CI, confidence interval


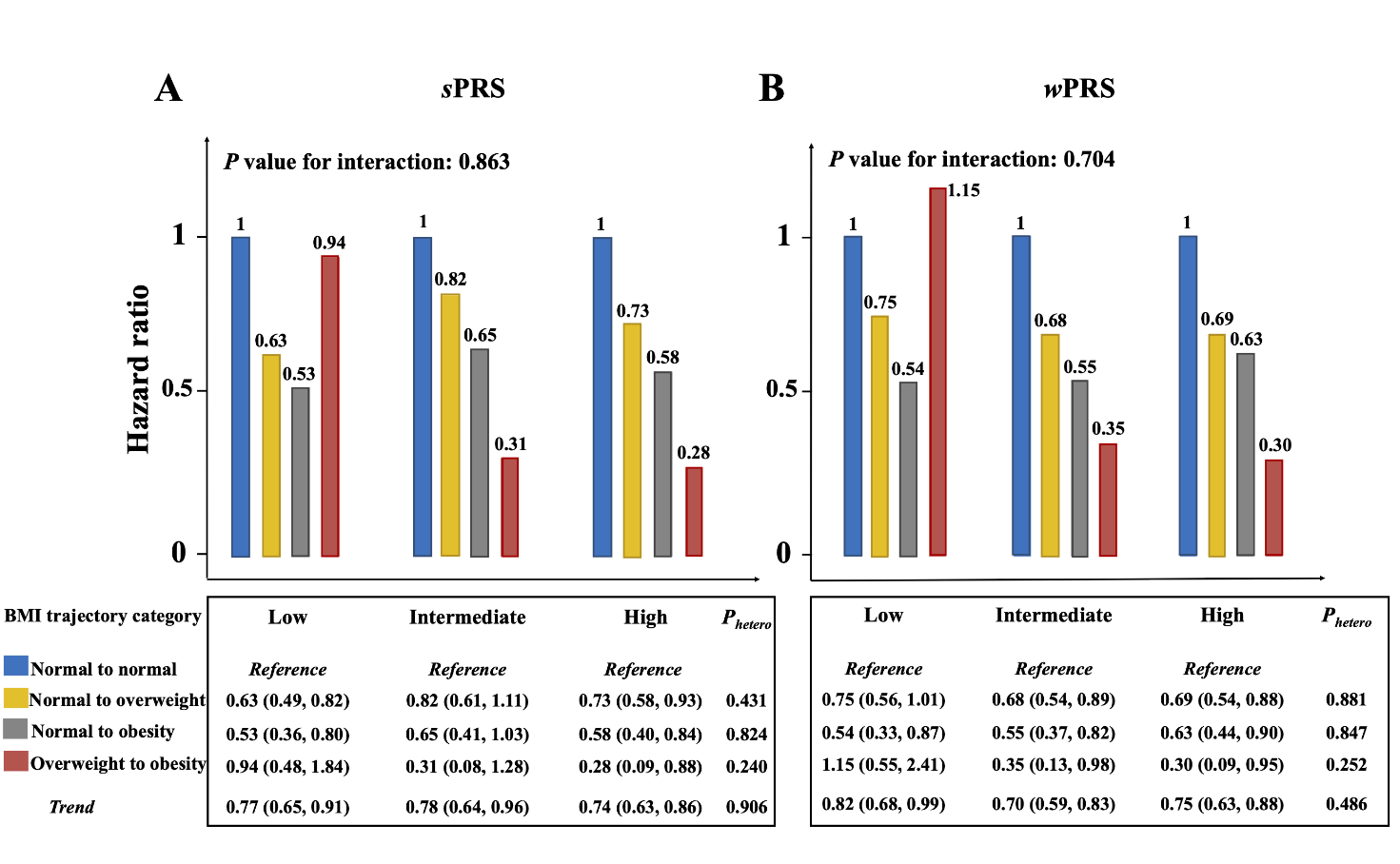


# **Figure S4. Association of multivariate-adjusted NSCLC risk with BMI trajectories according to PRS_GWIA_ categories.**

(a) *s*PRS_GWIA_ were calculated by simple counting. (b) *w*PRS_GWIA_ were weighted according to the strength of their association with lung cancer. P value for interaction was derived from multivariate-adjusted Cox proportional hazards regression model


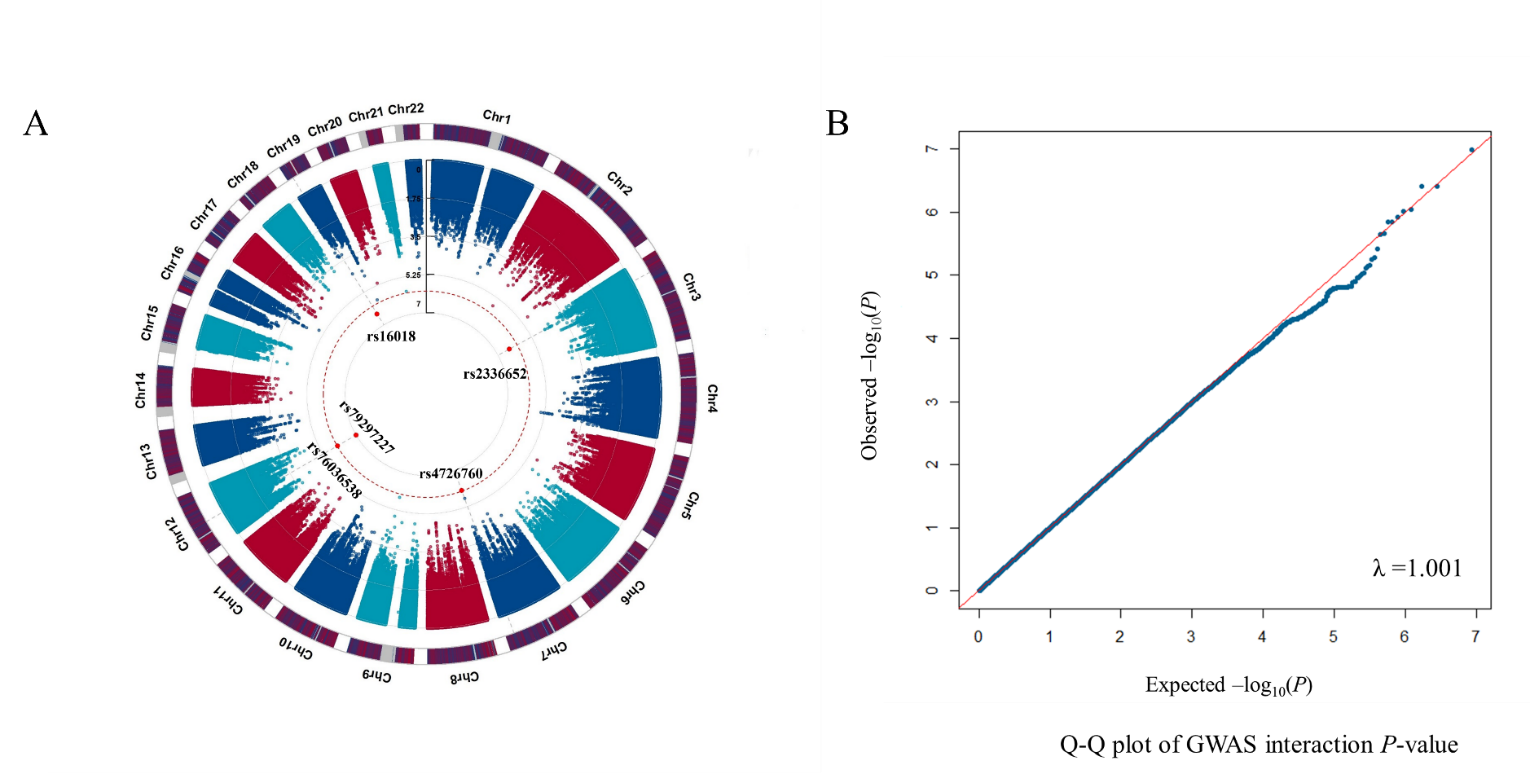


# **Figure S5. Circle Manhattan Plot for interaction analysis between SNPs and BMI trajectory regarding NSCLC risk.**

(A) X-axis shows chromosomal positions. Y-axis shows -log_10_*P* values for the interaction effect. The red circle dash line indicates preset threshold for *P* = 1×10^-6^. The grey circle solid line inside the red dash line indicates that the threshold reaches *P* = 1×10^-7^. (B) Q-Q plot of *P*-values from genome-wide gene-BMI trajectory interaction analyses

GWAS, Genome-wide association studies


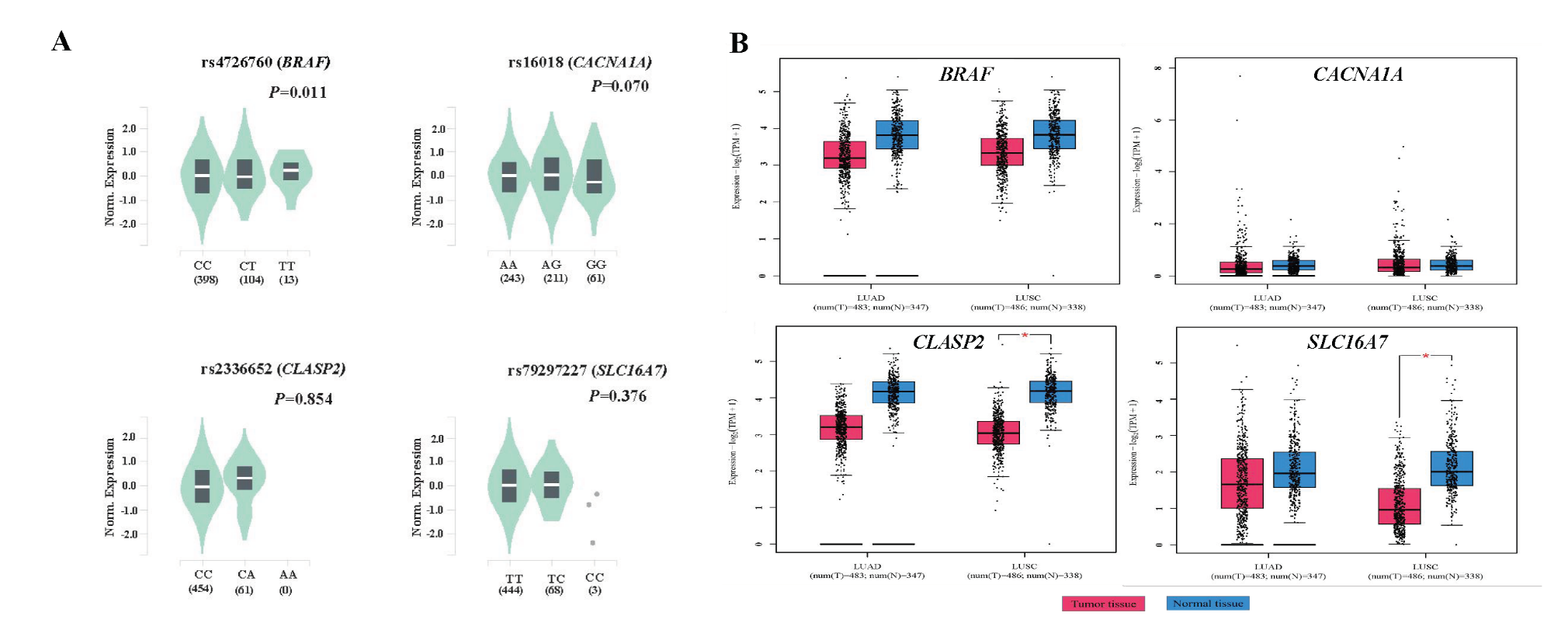


# **Figure S6. Analysis of the four loci and related gene expression in lung tissue.**

(A) Genotype of four GWIA-identified SNPs and related gene expression levels in the GTEx lung tissue. (B) The expression level of *BRAF*, *CACNA1A*, *CLASP2*, and *SLC16A7* mRNA in NSCLC and normal tissues (http://gepia2.cancer-pku.cn/#general). **P*< 0.05 compared with normal tissue
